# Early postnatal administration of an AAV9 gene therapy is safe and efficacious in CLN3 disease

**DOI:** 10.3389/fgene.2023.1118649

**Published:** 2023-03-24

**Authors:** Tyler B. Johnson, Jon J. Brudvig, Shibi Likhite, Melissa A. Pratt, Katherine A. White, Jacob T. Cain, Clarissa D. Booth, Derek J. Timm, Samantha S. Davis, Brandon Meyerink, Ricardo Pineda, Cassandra Dennys-Rivers, Brian K. Kaspar, Kathrin Meyer, Jill M. Weimer

**Affiliations:** ^1^ Pediatrics and Rare Diseases Group, Sanford Research, Sioux Falls, SD, United States; ^2^ Amicus Therapeutics, Cranbury, NJ, United States; ^3^ The Research Institute at Nationwide Children’s Hospital, Columbus, OH, United States; ^4^ Department of Pediatrics, Sanford School of Medicine, University of South Dakota, Vermillion, SD, United States

**Keywords:** neuronal ceroid lipofuscinosis, neurodegenerative disease, gene therapy, adeno-associated virus, rare disease

## Abstract

CLN3 disease, caused by biallelic mutations in the *CLN3* gene, is a rare pediatric neurodegenerative disease that has no cure or disease modifying treatment. The development of effective treatments has been hindered by a lack of etiological knowledge, but gene replacement has emerged as a promising therapeutic platform for such disorders. Here, we utilize a mouse model of CLN3 disease to test the safety and efficacy of a cerebrospinal fluid-delivered AAV9 gene therapy with a study design optimized for translatability. In this model, postnatal day one administration of the gene therapy virus resulted in robust expression of human *CLN3* throughout the CNS over the 24-month duration of the study. A range of histopathological and behavioral parameters were assayed, with the therapy consistently and persistently rescuing a number of hallmarks of disease while being safe and well-tolerated. Together, the results show great promise for translation of the therapy into the clinic, prompting the launch of a first-in-human clinical trial (NCT03770572).

## Introduction

Batten disease (or neuronal ceroid lipofuscinosis [NCL]) is a family of recessively inherited lysosomal storage disorders causing progressive neurological deficits, and, in most subtypes, premature death. This family of diseases is caused by mutations in one of at least 13 genes, some of which have been shown to have roles in lysosomal function and biogenesis (Johnson et al., 2019a; di Ronza et al., 2018). CLN3 Batten disease (CLN3 disease, also known as juvenile NCL) is caused by mutations in the ceroid lipofuscinosis neuronal 3 gene (*CLN3*). Patients typically present around the age of five with vision loss followed shortly thereafter by seizures and motor, cognitive, and behavioral deficits. The disease progresses rapidly, with premature death usually occurring around 20 years of age (Santavuori, 1988; Aberg et al., 2000).

CLN3 is known to localize to the lysosome and is necessary for efficient lysosomal function, but its precise molecular functions are unknown (Jarvela et al., 1999). Some lines of evidence suggest CLN3 may have unique roles in neurons, which are the primary cells affected in the disease (Rechtzigel et al., 2022). Disease causing mutations disrupt the trafficking of CLN3 into neuronal processes, where it colocalizes with synaptic markers (Jarvela et al., 1999; Luiro et al., 2001). Various neuronal deficits have been shown in *in vitro* models of the disease, including increased susceptibility to glutamatergic excitotoxicity (Kovacs et al., 2006; Finn et al., 2011), alterations in synaptic plasticity (Grünewald et al., 2017; Studniarczyk et al., 2018), impaired synaptogenesis (Gomez-Giro et al., 2019), and network dysfunction (Ahrens-Nicklas et al., 2019; Ahrens-Nicklas et al., 2022), but none of these have been linked directly to a molecular function for the protein. A recent study demonstrated that CLN3 regulates the retromer-dependent recycling of lysosomal sorting receptors (Yasa et al., 2020; Yasa et al., 2021), which could prove to be an important development for the field (Cotman and Lefrancois, 2021).

While etiology remains elusive, the pathological consequences of CLN3 deficiency have been well characterized in animal studies, mostly using the *Cln3* ^*−/−*^ and *Cln3^Δex7/8^* mouse models (Katz et al., 1999; Mitchison et al., 1999; Cotman et al., 2002; Eliason et al., 2007). These models consistently show pathological hallmarks of Batten disease, including lysosomal storage material accumulation (Mitchison et al., 1999; Cotman et al., 2002; Bosch et al., 2016), microglial activation (Weimer et al., 2009; Bosch et al., 2016), astrocytosis (Cotman et al., 2002; Weimer et al., 2009; Johnson et al., 2019b), and neurodegeneration (Pontikis et al., 2004; Pontikis et al., 2005; Weimer et al., 2007; Weimer et al., 2009), with storage accumulation becoming apparent at early time points around 3 months of age and the first evidence of glial activation and neurodegeneration appearing later, around five and 12 months of age, respectively (Pontikis et al., 2004; Pontikis et al., 2005). However, behavioral deficits in the *Cln3* mouse models have been difficult to replicate across different test sites, with phenotypes appearing to be highly sensitive to factors including genetic background and animal husbandry practices (Kovacs and Pearce, 2015; Timm et al., 2018; Johnson et al., 2019b)*.* Additionally, despite the often severe histopathology, lifespan is not reliably affected in *Cln3* ^*Δex7/8*^ mice (Cotman et al., 2002) and is only modestly reduced in *Cln3* ^*−/−*^ mice (Katz et al., 2008).

While these models have been utilized extensively to test potential therapies (Burkovetskaya et al., 2014; Aldrich et al., 2016; Palmieri et al., 2017; Dannhausen et al., 2018; Schultz et al., 2018; Tarczyluk-Wells et al., 2019), there remains no clinically accepted treatment that can halt disease progression (Johnson et al., 2019a). With only sparse knowledge of CLN3 function, therapies that could directly restore functional CLN3 are appealing options. Viral-delivered gene therapies have been explored preclinically for most forms of Batten disease (Johnson et al., 2019a), and clinically for CLN2, CLN3, CLN5, CLN6, and CLN7, with results pending (NCT#s: 01161576, 00151216, 01414985, 03770572, 04273243, 05228145, 04737460). In the CLN3 space, a recent preclinical study used an adeno associated virus serotype 9 (AAV9) vector to deliver human *CLN3* (*hCLN3*) to 1 month old *Cln3^Δex7/8^* mice (Bosch et al., 2016). The therapy reduced lysosomal storage material, glial activation, and motor deficits, suggesting that gene therapy could be an effective option for human CLN3 disease patients. However, the virus was delivered systemically through intravenous injection, which requires relatively high doses that have been linked to hepatotoxicity and sensory neuron degeneration (Hinderer et al., 2018). Moreover, intravenous delivery of AAV9 in 1-month old mice may not recapitulate the tropism/cellular targeting observed in non-human primates and presumed to occur in pediatric human patients (Chakrabarty et al., 2013; Schuster et al., 2014; Green et al., 2016). Additionally, outcomes were evaluated in male mice only and most were only tracked through 5 months of age, leaving unanswered questions about long-term safety and efficacy across both sexes.

Here, we present a long-term, translatable preclinical study of an AAV9 gene therapy for CLN3 disease. We tested the therapy in the *Cln3^Δex7/8^* mouse model, which recapitulates the most common mutation found in human CLN3 disease patients (Cotman et al., 2002). We delivered an AAV9 vector encoding human CLN3 (hCLN3) via a single intracerebroventricular (ICV) injection at postnatal day one (P1), based on our prior success with the same strategy in mouse models of CLN6 and CLN8 disease (Cain et al., 2019; Johnson et al., 2021). We conducted a comprehensive outcome assessment following a broad range of histopathological, hematologic, and behavioral markers through 24 months of age. We document a robust rescue of numerous disease correlates and confirm that the therapy is also safe and well tolerated. Collectively, this work provides strong support for the utility of such a strategy in the treatment of patients with CLN3 disease.

## Results

### P1 ICV administration of scAAV9.Mecp2.CLN3 achieves long-lasting expression of CLN3 throughout the brain

We designed a gene therapy vector to drive persistent, moderate-level expression of human CLN3 (hCLN3) throughout the brain. In this vector (scAAV9.Mecp2.CLN3) hCLN3 is expressed under the control of truncated Mecp2 promoter flanked by a modified simian vacuolating virus 40 (SV40) intron with a terminal bovine growth hormone (BGH) poly(A) signal. This vector is similar to the one currently being used in clinical trials for CLN6 disease patients (Cain et al., 2019), with the substitution of the more moderately expressing Mecp2 promoter for the previously used CMV enhanced chicken-β-actin (CB) promoter. This change in promoter has been found to be effective in a preclinical gene therapy study of CLN8 disease and is additionally congruent with the results of a recent preclinical study in which a similar human *MECP2* promoter was shown to be efficacious for AAV-mediated gene therapy in a *Cln3* mouse model (Bosch et al., 2016; Johnson et al., 2021).

**FIGURE 1:**
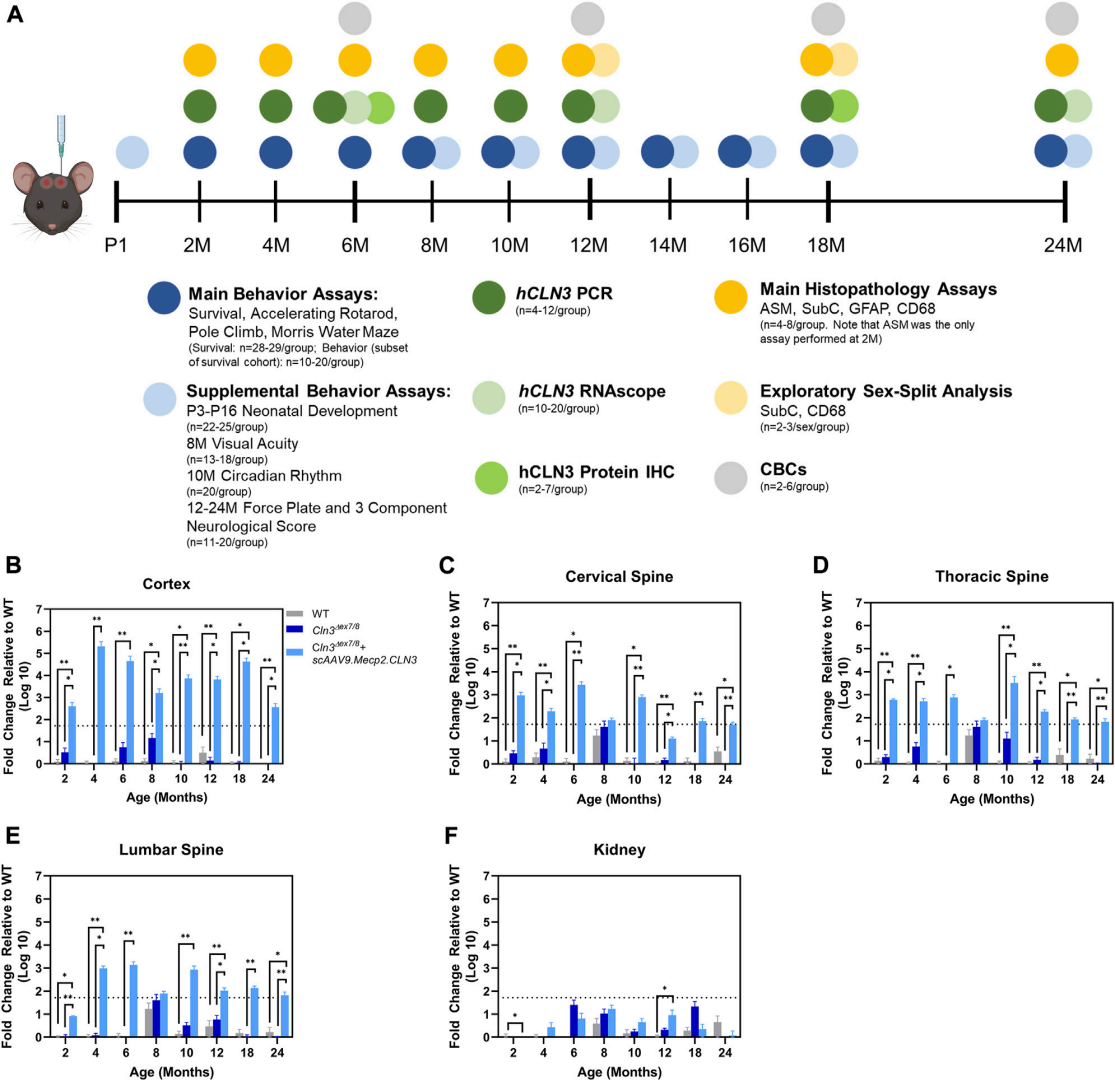
Overall study design and demonstration of *scAAV9.Mecp2.CLN3* administration resulting in hCLN3 expression throughout the brain and spinal cord. Overall study design **(A)**. N’s represent mixed and even sexes unless otherwise described. As measured by RT-qPCR, *hCLN3* is expressed in the cerebral cortex **(B)**, cervical **(C)**, thoracic **(D)**, and lumbar **(E)** spinal cord. *hCLN3* does not show strong expression in the kidney **(F)**. Kruskal-Wallis, Dunn correction at each time point, as each time point was processed independently. Mean ± SEM. *p < 0.05, **p < 0.01, ***p < 0.001, ****p < 0.0001. For A, mouse graphic obtained from BioRender.com.

To test the safety and efficacy of the therapy, we delivered 2.2 × 10^10^ vg of scAAV9.Mecp2.CLN3 via a single postnatal day one (P1) ICV injection in male and female *Cln3^Δex7/8^* mice. We followed three treatment groups for 24 months (C57BL/6 wild type, *Cln3^Δ7/8^* + PBS, and *Cln3^Δ7/8^* + scAAV9.Mecp2.CLN3; n = 28–29 mice/group, mixed and even sexes), over which time the treatment was generally well-tolerated. A subset of the survival cohort was examined in neurobehavior tests from 2 to 24 months of age (Figure 1A, n = 10–20/group, mixed and even sexes), and as behavior assessments in the *Cln3^Δ7/8^* mouse model have been notoriously inconsistent, two additional cohorts were examined for exploratory behavior tests outside of the main behavior group. This included a group of neonatal mice that were examined for neonatal reflex development (n = 22–25/group, mixed and even sexes) and a group of adult mice that were examined for visual acuity at 8 months of age and circadian rhythm at 10 months of age (n = 13–20/group, mixed and even sexes). Lastly, individual cohorts of animals were assessed for gene expression and histopathology primarily at 2, 4, 6, 8, 10, 12, 18, and 24 months of age (n = 4–8/group, mixed and even sexes), with a few exploratory assessments performed outside of these time points.

**FIGURE 2:**
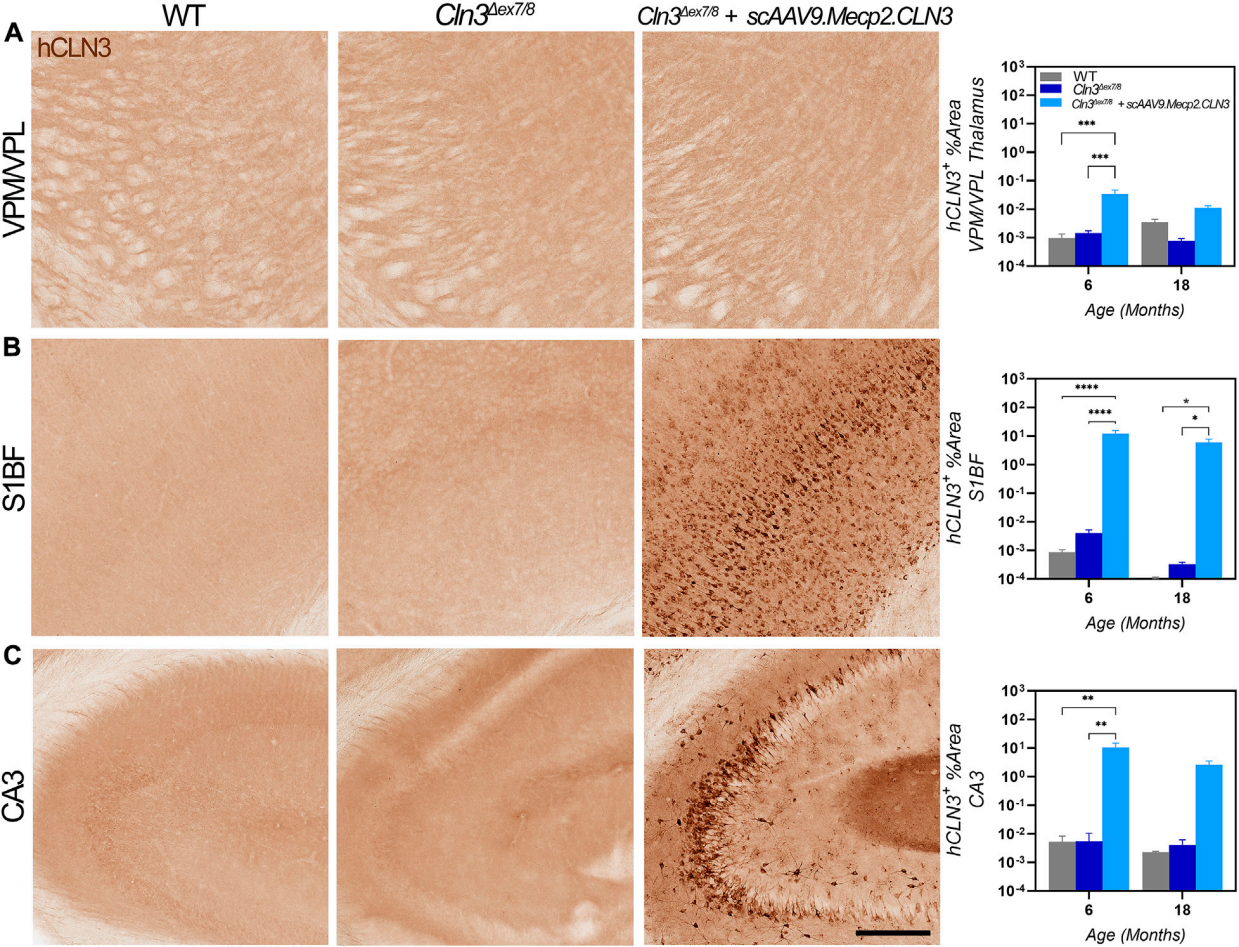
hCLN3 protein is expressed in the brains of *scAAV9.Mecp2.CLN3* treated *Cln3* ^*Δex7/8*^ mice. AAV9 administration generates hCLN3 protein expression in the thalamus (VPM/VPL, **(A)**, somatosensory cortex (S1BF; **(B)**, and CA3 of the hippocampus **(C)**, most notably at 6 months of age. Two-way ANOVA, Tukey correction. Mean ± SEM. *p < 0.05, **p < 0.01, ***p < 0.001, ****p < 0.0001. Scale Bar = 200 µm, images presented are from 6 months of age. 12 images/region/animal were examined.

AAV9-delivered transgenes have been shown to target brain neurons with high efficiency, but existing studies have administered the virus between one and 2 months of age using vascular or direct brain parenchymal injection (Garg et al., 2013; Bosch et al., 2016). Our prior work with CLN6 and CLN8 diseases (Cain et al., 2019; Johnson et al., 2021) has demonstrated that the ICV route of administration can be utilized efficaciously with substantially lower doses of virus (20- to 40-fold lower than the aforementioned studies using the AAV9-Mecp2 combination) while reducing potential toxicity issues encountered with high intravenous doses and the local trauma created by parenchymal injections (Garg et al., 2013; Bosch et al., 2016). Additionally, administration at P1 in mice best recapitulates the transduction patterns observed in non-human primates and presumed to occur in human patients (Chakrabarty et al., 2013; Schuster et al., 2014; Green et al., 2016). To confirm that ICV administration achieved persistent, widespread transduction in brain regions that are susceptible in CLN3 disease, including the somatosensory cortex (S1BF) and ventral posteromedial/lateral nuclei (VPM/VPL) of the thalamus, we examined hCLN3 transcript levels qualitatively from six to 24 months of age using RNAScope, a modified in situ hybridization technique. Transgene transcription was evident in every brain region that was examined beginning at 6 months, with the greatest proportion of expressing cells in the cerebral cortex (motor cortex, S1BF, and visual cortex), and lower levels of expression in the VPM/VPL, pons, cerebellum, and medulla (Supplementary Figure S1). Consistent and robust expression was maintained in all regions through 24 months. We also examined *hCLN3* transcript abundance with RT-qPCR. Transcript was evident in the cerebral cortex, cervical, thoracic, and lumbar spinal cord at most time points beginning at 2 months of age but was absent in the kidney (Figures 1B–F). Lastly, hCLN3 protein levels were measured in three different brain regions by standard immunohistochemistry. hCLN3 protein expression was evident in all three regions examined at 6 months of age, particularly in the S1BF and cornu ammonis 3 region (CA3) of the hippocampus (Figure 2).

### scAAV9.Mecp2.CLN3 rescues histopathological hallmarks of disease in *Cln3^Δex7/8^* mice

To investigate whether restoration of hCLN3 prevents brain pathology, we examined various markers of disease burden from two to 24 months of age. Since *Cln3^Δex7/8^* mice have a relatively mild neurodegeneration phenotype (Pontikis et al., 2004), we focused on markers that appear early, show progressive changes, and have strong face validity for monitoring disease progression.

**FIGURE 3:**
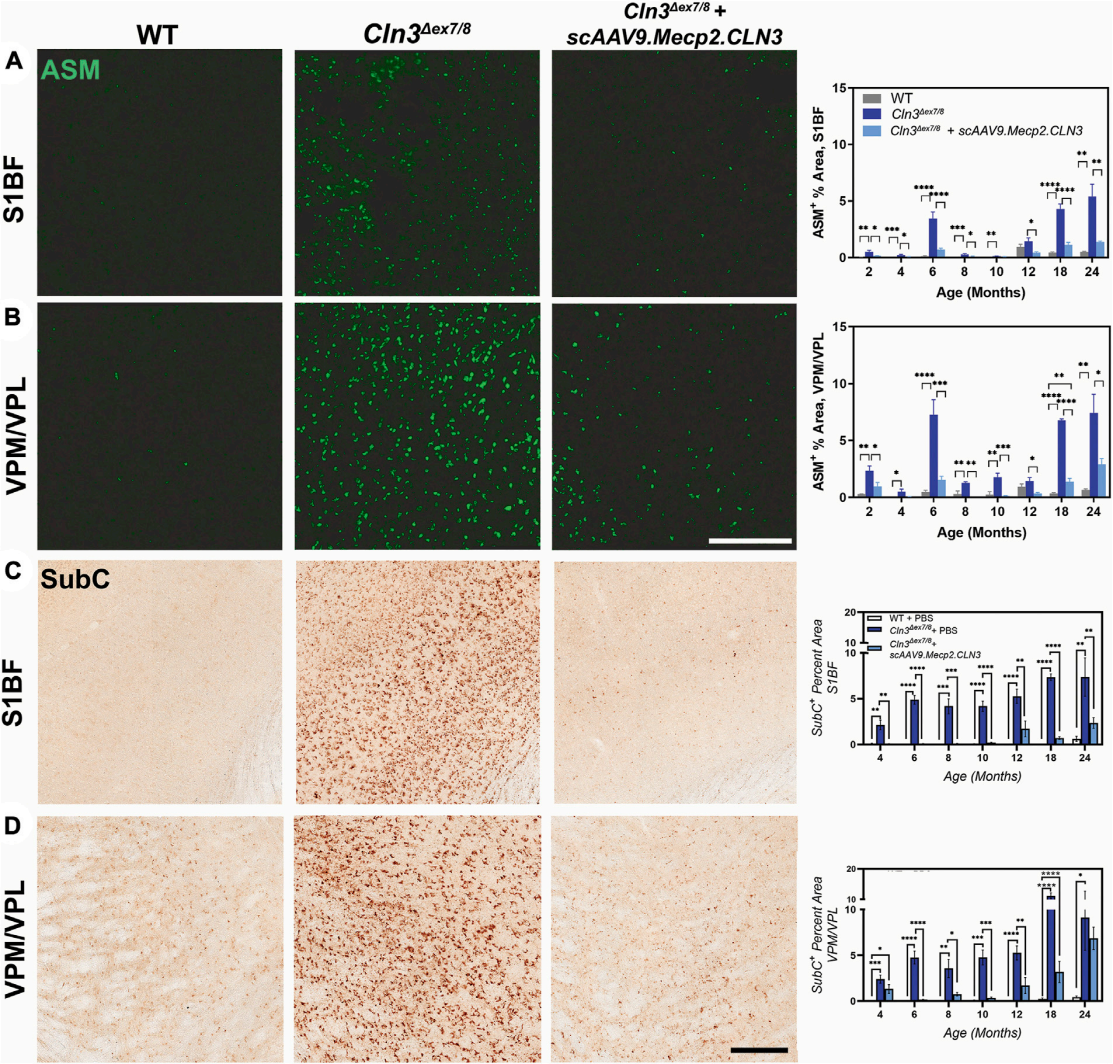
*scAAV9.Mecp2.CLN3* administration attenuates storage material accumulation in *Cln3* ^*Δex7/8*^ mice. AAV9 administration attenuates autofluorescent storage material accumulation in the S1BF **(A)** and VPM/VPL **(B)** from 2 to 24 months of age in *Cln3* ^*Δex7/8*^ mice. Similarly, AAV9 administration attenuates the accumulation of a lipofuscin constituent, mitochondrial ATP synthase subunit c (SubC) in the S1BF **(C)** and VPM/VPL **(D)** throughout the lifespan of *Cln3* ^*Δex7/8*^ mice. One-way ANOVA, Tukey correction at each time point, as each time point was processed independently. Mean ± SEM. *p < 0.05, **p < 0.01, ***p < 0.001, ****p < 0.0001. Scale Bars = 200 µm, images presented are from 6 months of age. 8 images/region/animal were examined for ASM; 12 images/region/animal were examined for SubC.

The accumulation of autofluorescent storage material (ASM) in lysosomes is a well-established NCL disease marker. In *Cln3^Δex7/8^* mice, ASM is evident in a variety of tissues and cell types but is particularly pronounced in cortical and thalamic neurons (Cotman et al., 2002). While it is unclear if the presence of ASM contributes towards cellular dysfunction and degeneration, its ubiquity and early appearance in NCL suggests that it is at least a correlate of disease initiation and progression. We examined ASM in the S1BF and in the VPM/VPL from two to 24 months of age and observed striking differences in treated versus untreated mice (Figures 3A, B). ASM was already evident in the S1BF and VPM/VPL at 2 months of age in PBS-treated *Cln3^Δex7/8^* mice and remained elevated through 24 months of age. Treatment with scAAV9.Mecp2.CLN3 reduced ASM in both regions and treated animals were generally indistinguishable from wild type animals until 18 months of age.

Lysosomal dysfunction can also be observed by examining lysosomal accumulation of mitochondrial ATP synthase subunit C (SubC), a major constituent of the storage material in multiple NCLs (Palmer et al., 1992; Cotman et al., 2002). We examined SubC immunoreactivity in the S1BF and VPM/VPL with findings similar to those for ASM (Figures 3C, D); SubC immunoreactivity was elevated in both regions in PBS-treated *Cln3^Δex7/8^* mice from two to 24 months of age and was reduced by treatment with scAAV9.Mecp2.CLN3. Again, SubC immunoreactivity in scAAV9.Mecp2.CLN3-treated *Cln3^Δex7/8^* mice was in the same range as wild type animals until approximately 12 months of age. Combined, the reductions in ASM and SubC suggest that scAAV9.Mecp2.CLN3 is able to restore at least some lysosomal function in *Cln3^Δex7/8^* mice.

**FIGURE 4:**
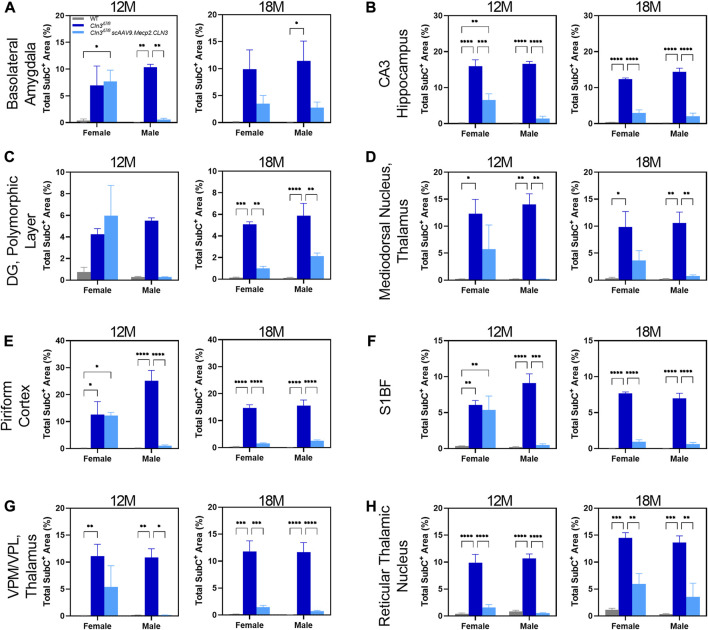
Transient, male specific responses in AAV9 mediated Subunit C prevention in novel pathological brain regions. Previously undescribed Subunit C accumulation was found in the basolateral amygdala **(A)**, CA3 of the hippocampus **(B)**, dentate gyrus polymorphic layer **(C)**, mediodorsal nucleus of the thalamus **(D)**, piriform cortex **(E)**, and reticular thalamic nucleus **(H)**. Additionally, there was a male specific response in AAV9 prevention of Subunit C accumulation in every area examined at 12 months of age, including in the previously described S1BF **(F)** and VPM/VPL **(G)**. This male specific response was transient and was not present at 18 months of age **(A–H)**. Two-way ANOVA, Tukey correction at each time point. Mean ± SEM. *p < 0.05, **p < 0.01, ***p < 0.001, ****p < 0.0001. 12 images/region/animal were examined.

As sex-specific responses to gene therapy treatment in Batten disease are largely unknown, we performed a preliminary, sex-split analysis of our SubC data at 12 and 18 months of age to understand whether there are long-term, sustained differences in gene therapy response between the sexes. Interestingly, we observed significant sex differences in terms of the SubC treatment response in some brain regions and time points. At 12 months of age, scAAV9.Mecp2.CLN3 treated *Cln3^Δex7/8^* males exhibited a profound reduction in SubC immunoreactivity in several brain regions that was not evident in females (Figure 4). Sex differences were not evident at 18 months, with both sexes exhibiting a robust response to scAAV9.Mecp2.CLN3 treatment, indicating that any male-specific sex differences are transient.

**FIGURE 5:**
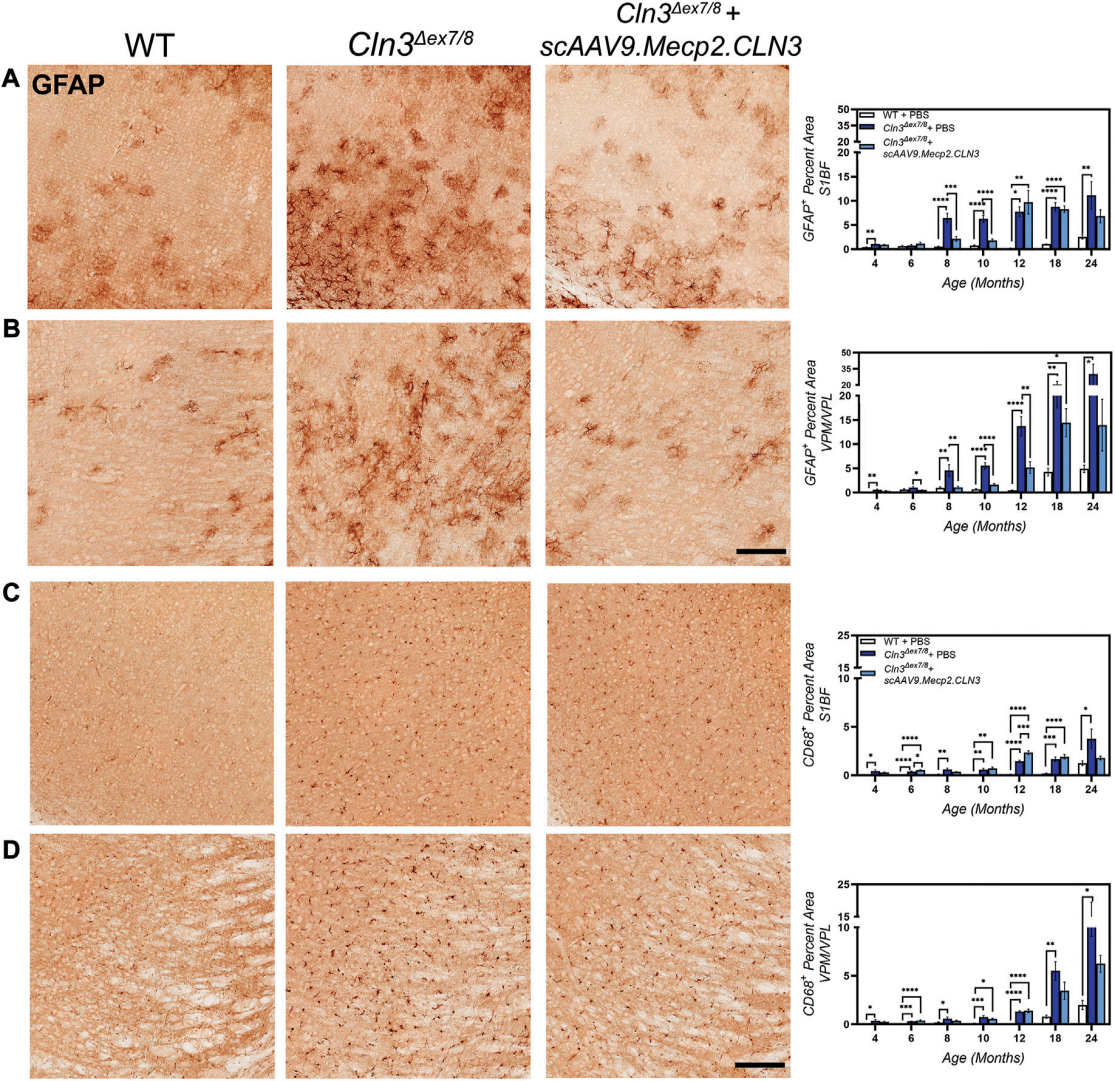
*scAAV9.Mecp2.CLN3* reduces glial activation in *Cln3* ^*Δex7/8*^ mice. Neonatal administration of AAV9 reduces astrocyte activation (GFAP immunoreactivity) at several time points in the S1BF **(A)** and VPM/VPL **(B)** of *Cln3* ^*Δex7/8*^ mice. AAV9 also reduces microglial activation (CD68 immunoreactivity) in the S1BF **(C)** and VPM/VPL **(D)** of *Cln3* ^*Δex7/8*^ mice at several time points. Scale Bars = 25 µm, images presented are from 8 months of age. One-way ANOVA, Tukey correction at each time point, as each time point was processed independently. Mean ± SEM. *p < 0.05, **p < 0.01, ***p < 0.001, ****p < 0.0001. 12 images/region/animal were examined.

Neuroinflammation, as evidenced by activated astrocytes and microglia, is another early hallmark of CLN3 disease in animal models, preceding neurodegeneration (Cotman et al., 2002; Pontikis et al., 2004). To investigate whether scAAV9.Mecp2.CLN3 could attenuate neuroinflammation in *Cln3^Δex7/8^* mice, we examined markers of astrocytic and microglial activation. In neurodegenerative disease states, astrocytes respond to environmental cues by upregulating the expression of glial fibrillary acidic protein (GFAP) (Liddelow and Barres, 2017). In the S1BF and VPM/VPL of PBS-treated *Cln3^Δex7/8^* mice, we observed hypertrophic and intensely GFAP-positive astrocytes, with increases in GFAP immunoreactivity beginning at 4 months of age (Figures 5A, B). In the S1BF, GFAP immunoreactivity peaked at 10–12 months and persisted through 24 months, while in the VPM/VPL GFAP immunoreactivity peaked at 24 months of age. Treatment with scAAV9.Mecp2.CLN3 did not have a large effect on GFAP-immunoreactivity in the S1BF, but treatment did reduce GFAP-immunoreactivity in the VPM/VPL at four, 10, and 12 months of age. At most early time points in the VPM/VPL, GFAP-immunoreactivity levels in scAAV9.Mecp2.CLN3 treated mice were indistinguishable from wild type.

Patterns of disease progression were also evident for activated microglia, as evidenced by changes in CD68 immunoreactivity (Figures 5C, D). Microglia in PBS-treated *Cln3^Δex7/8^* mice were hypertrophic and intensely CD68-positive, indicative of an activated state. CD68 immunolabeling was increased in PBS-treated *Cln3^Δex7/8^* mice from four to 18 months in the S1BF and VPM/VPL, with peak levels occurring at 24 months of age. Treatment with scAAV9.Mecp2.CLN3 reduced CD68 immunoreactivity at later stages of disease progression (i.e. 18 and 24 months), but did not appear to have a large effect at earlier time points.

**FIGURE 6:**
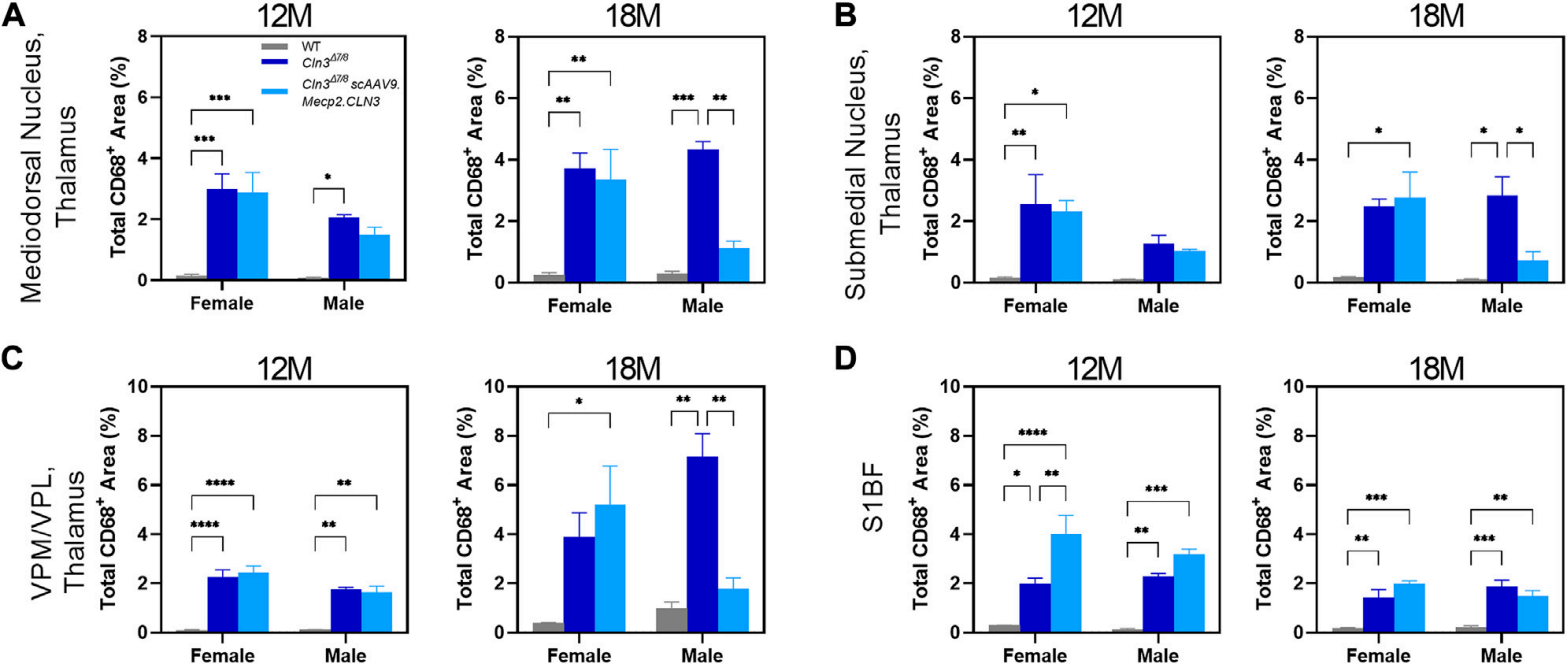
Transient, male-specific responses in AAV9 mediated microglial suppression. Previously undescribed microglial activation was found in the mediodorsal nucleus of the thalamus **(A)** and submedial nucleus of the thalamus **(B)** at 12 months of age. Additionally, there was a male specific response in AAV9-mediated reduction of microglial activation at 18 months of age in these two regions, as well as in the previously described VPM/VPL **(C)**. There were no sex differences detected in the S1BF **(D)**. Two-way ANOVA, Tukey correction at each time point. Mean ± SEM. *p < 0.05, **p < 0.01, ***p < 0.001, ****p < 0.0001. 12 images/region/animal were examined.

As for SubC, we performed a preliminary, sex-split analysis of our CD68 data at 12 and 18 months. We observed significant sex differences in the CD68 treatment response in some brain regions and time points (Figure 6). At 12 months of age, scAAV9.Mecp2.CLN3 treatment did not reduce CD68 immunoreactivity in either sex in the brain regions examined. However, at 18 months of age, scAAV9.Mecp2.CLN3 treatment significantly reduced CD68 immunoreactivity in male *Cln3^Δex7/8^* mice in three of the four regions examined, while female mice were unaffected. These perplexing sex differences are not easily explained by the available data, but suggest a need for further inquiry in future studies with larger cohorts of animals.

Overall, scAAV9.Mecp2.CLN3 treatment achieved widespread and persistent reductions in histopathological hallmarks of brain disease in *Cln3^Δex7/8^* mice. In an attempt to detect changes in peripheral markers of disease, which have been shown to be highly sensitive to environmental factors in *Cln3^Δex7/8^* mice (Timm et al., 2018), we also collected blood samples at six, 12, 18, and 24 months of age and performed complete blood counts (CBCs, Supplementary Figure S2). The only difference that was detected was a minor, transient reduction in hemoglobin levels in *Cln3^Δex7/8^* mice at 6 months of age that was completely prevented by treatment with scAAV9.Mecp2.CLN3.

### scAAV9.Mecp2.CLN3 treatment shows benefits in behavioral parameters in *Cln3^Δex7/8^* mice

While histopathological hallmarks of disease manifest reliably in *Cln3^Δex7/8^* mice, other phenotypes are known to be more difficult to replicate as they are highly sensitive to factors such as genetic background and animal husbandry practices (Kovacs and Pearce, 2015; Timm et al., 2018; Johnson et al., 2019b). We therefore employed a comprehensive battery of motor, visual, and behavioral assays to investigate the functional consequences of scAAV9.Mecp2.CLN3 treatment in *Cln3^Δex7/8^* mice.

**FIGURE 7:**
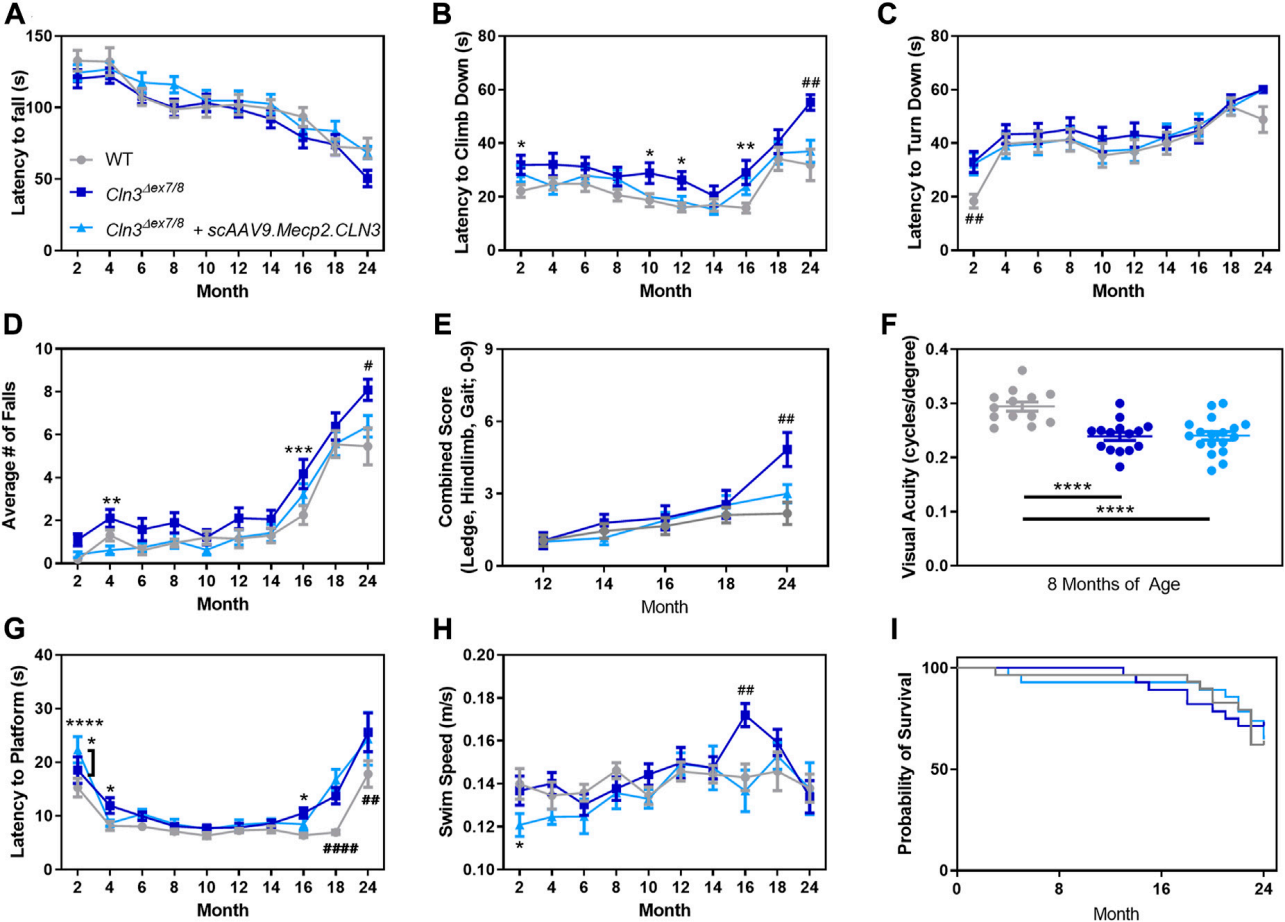
AAV9 administration prevents late stage motor deficits, but not memory/learning or visual deficits. While *Cln3* ^*Δex7/8*^ mice do not show strong behavioral deficits in the accelerating rotarod **(A)**, AAV9 administration prevented motor deficits at 24 months of age in the pole climb descent **(B)**, fall **(D)**, and neurodegeneration score **(E)**. AAV9 administration does not prevent early motor presentations in pole climb turn **(C)**, visual deficits as measured by optokinetic tracking **(F)**, or memory/learning deficits as measured by a Morris water maze **(G,H)**. No survival differences were detected between the three treatment groups **(I)**. Two-way ANOVA, Tukey correction **(A–E,G,H)**, One-way ANOVA, Tukey correction **(F)**, and Mantel-Cox log rank. Mean ± SEM. Asterisks denote comparison to wild type unless otherwise noted. Hash signs denote comparison to all other groups. *p < 0.05, **p < 0.01, ***p < 0.001, ****p < 0.0001. #*p* < 0.05, ##*p* < 0.01, ###*p* < 0.001, ####*p* < 0.0001. Detailed Ns in Supplementary Table S2.

To investigate whether motor coordination deficits in *Cln3^Δex7/8^* mice are affected by scAAV9.Mecp2.CLN3 treatment, we utilized the rotarod test, pole climb test, and a three component neurological score that has shown utility in similar disease models (Guyenet et al., 2010; Cain et al., 2019). The rotarod test revealed no clear differences between groups, with the three groups exhibiting similar performance at all time points (Figure 7A). Disease impact and treatment effect were evident in the pole climb results with PBS-treated *Cln3^Δex7/8^* mice exhibiting increased latency to climb downwards at two, 10, 12, 16, and 24 months (Figure 7B) and an increased number of falls from the pole climb apparatus at four, 16, and 24 months (Figure 7D). At all of these time points, scAAV9.Mecp2.CLN3 treated animals performed at levels indistinguishable from wild type. A clear phenotype was also observed at 24 months in the three component test, with PBS-treated *Cln3^Δex7/8^* mice exhibiting increased combined scores (ledge descent ability, hind limb clasping, and gait), and scAAV9.Mecp2.CLN3 treatment rescuing scores to levels indistinguishable from wild type (Figure 7E).

We additionally examined locomotor activity patterns using a force plate actimeter from 12–24 months of age. Body weight as measured by the force plate was similar between the three groups at most time points, with the exception of 24 months weight that was lower in PBS-treated *Cln3^Δex7/8^* mice but sustained in scAAV9.Mecp2.CLN3 treated animals (Supplementary Figure S3A). Distance traveled, area traveled, bouts of low mobility, and focused stereotypy events followed a similar pattern, with 18–24 month old PBS-treated *Cln3^Δex7/8^* mice showing hyperactivity that was generally prevented by scAAV9.Mecp2.CLN3 treatment (Supplementary Figures S3B–E). We considered the possibility that other tests might be able to detect differences in motor function at earlier time points, but a collection of neonatal tests found no differences between treatment groups over P3-P16 (Supplementary Figure S4).

In order to investigate potential deficits in visual acuity, learning, and memory, we tested mice in a visual-cued Morris water maze. First, we measured visual acuity directly using optokinetic tracking at a single time point. At 8 months of age, PBS-treated mice exhibited decreased visual acuity and scAAV9.Mecp2.CLN3 had no appreciable effect on this phenotype (Figure 7F). In the Morris water maze, some minor differences in platform finding latency were evident at later time points beginning at 16 months of age, with scAAV9.Mecp2.CLN3 treatment having no benefit from 18 to 24 months of age (Figures 7G, H). However, the results were confounded by the visual acuity results along with differences in swim speed at 16 months of age, making it difficult to ascertain whether scAAV9.Mecp2.CLN3 treatment had any direct effect on memory/learning deficits.

**FIGURE 8:**
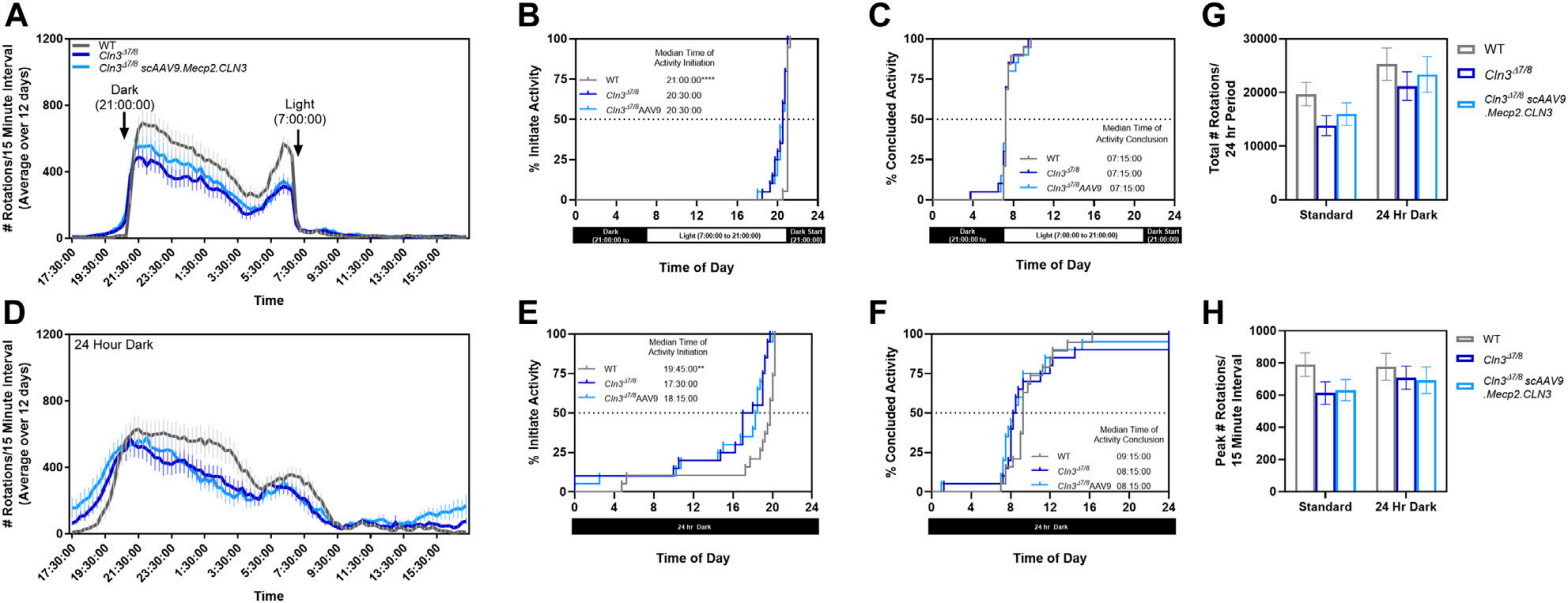
*Cln3^Δex7/8^* mice show altered circadian activity patterns. When measured in a home cage running wheel apparatus during a regular light/dark cycle **(A)**, 10-month old *Cln3^Δex7/8^* mice of both treatment groups are active significantly earlier than their wild type counterparts **(B,C)**. When measured during a 24 hour dark cycle **(D)**, 10-month old *Cln3^Δex7/8^* mice of both treatment groups are also active significantly earlier than their wild type counterparts **(E,F)**. The total number of rotations **(G)** and peak number of rotations **(H)** were not altered between groups. For **(B,C,E,F)**: log-rank Mantel-Cox, Bonferroni post-hoc; For **(G,H)**: One-way ANOVA within each test cycle, Tukey post-hoc. Mean ± SEM. **p < 0.01, ****p < 0.0001.

As sleep disturbance is a common issue experienced by individuals affected by CLN3 disease, we sought to explore whether there were differences in circadian activity patterns in our mice. Day and night wheel running behavior was quantified over 12 days at a single time point (10 month old mice). When monitored using our facility’s normal light-dark cycle (14 h light, 10 h dark from 21:00 to 7:00), *Cln3^Δex7/8^* mice were active significantly earlier than their wild type counterparts (Figures 8A–C). Specifically, *Cln3^Δex7/8^* mice regardless of treatment began activity about 30 min earlier than wild type mice (20:30 vs 21:00).

When monitored using a 24 h dark cycle to detect activity differences free from external light cues, wild type animals began activity approximately 1.25 h earlier than normal (19:45 vs 21:00) while *Cln3^Δex7/8^* mice began activity approximately two to 3 hours earlier than normal (17:30–18:15 vs 20:30) (Figures 8D–F). While *Cln3^Δex7/8^* mice were active for longer periods of time, this did not result in more rotations overall or more intense activity bursts (Figures 8G, H).

Lastly, survival was assessed from two to 24 months of age with no differences detected among treatment groups, recapitulating known limitations of the *Cln3^Δex7/8^* model (Cotman et al., 2002) (Figure 7I, Supplementary Table S1). We observed several deaths in each treatment group throughout the study, which were not significantly different between groups. Of the nine deaths among scAAV9.Mecp2.CLN3 treated *Cln3^Δ7/8^* animals, five were found dead, one was euthanized at 4 months of age for a possible urethral obstruction, one was euthanized at 5 months of age for hunched posture, one was euthanized at 21 months of age due to a facial mass, and one was euthanized at 24 months of age due to a persistent wound. Five PBS-treated *Cln3^Δ7/8^* animals and four wild type animals were also found dead during the course of the study, and one PBS-treated *Cln3^Δ7/8^* animal was euthanized at 21 months of age due to a mass on the shoulder/throat. Overall, while the behavioral data reflected the difficulties of working with the *Cln3^Δex7/8^* model, clear differences were evident in multiple assays at several time points, with scAAV9.Mecp2.CLN3 providing clear benefit.

## Discussion

Batten disease has been a challenging disease to treat due to a lack of etiological understanding. While great strides are being made towards understanding the functions of CLN proteins, affected patients and families are in immediate need for treatments that can combat disease progression. Thankfully, gene therapy has emerged as a powerful therapeutic tool in diseases such as these, where restoration of functional disease proteins in vulnerable cell populations can restore disrupted cellular processes and prevent downstream pathology. Recent work has shown that CSF-delivered AAV gene therapies may be safe and effective for CLN1 (Rozenberg et al., 2016; Shyng et al., 2017; Rozenberg et al., 2018), CLN2 (Katz et al., 2015), CLN3 (Bosch et al., 2016), CLN5 (Mitchell et al., 2018), CLN6 (Cain et al., 2019), CLN7 (Chen et al., 2020; Chen et al., 2022), and CLN8 (Johnson et al., 2021) diseases. Here, we describe an optimized gene therapy approach for CLN3 disease, which rescues numerous histopathological and behavioral correlates of disease.

We performed a one-time ICV delivery of an AAV9 vector driving expression of hCLN3 with a truncated Mecp2 promoter (scAAV9.Mecp2.CLN3) in neonatal *Cln3^Δex7/8^* mice. This strategy achieved widespread and long-term expression of hCLN3 transcript and protein throughout the CNS with some of the most abundant signal in the cerebral cortex. Restoration of CLN3 resulted in potent and long-lasting reductions in lysosomal storage burden, as measured by two separate markers. Neuroinflammation as quantified by GFAP immunoreactivity was also prevented in a subset of brain regions through 12 months of age. While other CNS routes of administration were considered (i.e., lumbar puncture (LP) or intracisterna magna (ICM)), the technical difficulty of the injection techniques in mice precluded these routes of administration (ROA). All three of these CNS ROAs, however, result in lower levels of transduction of the deeper brain structures (putamen, caudate, thalamus, striatum, and deep cerebellar nuclei) of non-human primates, and numerous studies have evaluated various ROAs for AAV delivery. Regardless of the variability between various studies, the general conclusion is that no CNS-targeted ROA provides superior biodistribution and that the decision on injection route for CNS disorders, in a clinical setting, should be determined through surgical/anatomical considerations (Ohno et al., 2019; Bailey et al., 2020; Kondratov et al., 2021; Meseck et al., 2022; Chen et al., 2023). Additionally, while our study did not examine the efficacy of gene therapy in post-symptomatic animals, several studies have shown that the greatest efficacy for CNS and other lysosomal storage disorders is achieved as early as possible, including in Batten disease (Cabrera-Salazar et al., 2007; Fu et al., 2016; Chen et al., 2022).

We observed unexpected spatiotemporal sex differences in the histopathological treatment response, with scAAV9.Mecp2.CLN3-treated males more consistently exhibiting reductions in SubC and CD68 immunoreactivity. While we have observed phenotypic sex differences in other models of NCL (Kovacs and Pearce, 2015; Poppens et al., 2019), this is the first time that differences have been observed specifically in terms of a gene therapy treatment response in this family of diseases. It remains to be seen how relevant these sex differences are to human patients, but these findings call into question the historical practice of using only male mice for preclinical NCL studies (McShane and Mole, 2022). However, it is plausible that the selective pathological accumulation in various regions described (Figures 4, 6) and the inability to fully correct pathological phenotypes in those regions play a role in altered visual processing (Figure 7F), changes in wakefulness/circadian rhythm (Figure 8), and memory/learning capabilities (Figure 7G). For example, despite transducing the hippocampus, prominent SubC accumulation persists in the polymorphic layer of the dentate gyrus (Figure 4C) at 18 months of age, which also coincides with increased latency to platform in Morris water maze in *Cln3^Δex7/8^* mice regardless of treatment (Figure 7G), consistent with memory and learning deficits resulting from hippocampal pathology. Further, as our results were examined in an exploratory light, future studies should examine the influence of sex on gene therapy response in more critical detail. Specifically, our experiments examining sex differences were preliminarily and were not sufficiently powered, thus future studies will need to be conducted to determine if these results are consistent in a larger cohort of animals. Additionally, we only examined sex differences in selected brain regions at limited time points (12 and 18 months of age), and future studies should examine the time course of any sex differences throughout the brain in greater detail.

We also assessed treatment outcomes using a comprehensive battery of motor, visual, and behavioral tests. It proved difficult to detect early and persistent disease changes in the *Cln3^Δex7/8^* model, but motor challenges revealed several important phenotypes that we were able to assess for treatment effects. PBS-treated *Cln3^Δex7/8^* mice exhibited motor deficits in the pole climb test at several time points, with scAAV9.Mecp2.CLN3 treatment consistently rescuing this to wild type levels. Similarly, PBS-treated *Cln3^Δex7/8^* mice had increased 24-month scores in a three-component test of motor function, and scAAV9.Mecp2.CLN3 provided a robust rescue.

Importantly we reported on a newly described phenotype in *Cln3^Δex7/8^* mice. Sleep disturbance is reported in a majority of CLN3 patients and is a significant contributor to reduced quality of life at various stages of disease progression. Numerous areas of sleep quality are affected with the primary complains being nightmares, reduced total sleep time, and fragmented sleep resulting in multiple awakenings that increase with progression of the disease (Ostergaard, 2016). Additionally, changes in circadian rhythms have been described at advanced stages of disease (Heikkila et al., 1995). We explored circadian rhythm patterns in *Cln3^Δex7/8^* mice and the effect of treatment on resulting behaviors utilizing spontaneous running wheels to measure wakefulness and levels of activity. Interestingly, *Cln3^Δex7/8^* mice were active significantly earlier than their wild type counterparts, and while *Cln3^Δex7/8^* mice were active for longer periods of time, activity levels were reduced compared to control mice (decreased number of rotations). It is likely that a combination of functional, psychological, and possibly pain-related phenotypes play a role in sleep disturbances in patients. However, regional pathological deficits in the CNS may also play a role in disrupted circadian rhythm and overall sleep quality. For instance, *Cln3^Δex7/8^* mice show selective SubC accumulation in the reticular nucleus of the thalamus (Figure 4H) and the habenular nuclei (data not shown), both of which have critical roles in the brain’s intrinsic circadian timekeeping and sleep processes (Bano-Otalora and Piggins, 2017; Vantomme et al., 2019). Although this newly discovered mouse behavior was not rescued with this therapy, it may be useful in future studies to evaluate potential drug targets’ ability to rescue sleep disturbances that are disruptive to the quality of life of patients. Unfortunately, the *Cln3^Δex7/8^* mouse model does not reliably recapitulate the reduced lifespan observed in human CLN3 patients (Cotman et al., 2002), and we observed no differences in lifespan between the three groups in our study. We did, however, perform extensive necropsy at each tissue collection time point through the advanced age of 24 months and observed no abnormalities specifically related to scAAV9.Mecp2.CLN3 treatment. Future studies could build on these results by testing in more severe models, such as the *Cln3^Δex7/8^*, Δex7/8:hAPP model, which exacerbated the CLN3 phenotype by introducing one copy of a human amyloid precursor protein (hAPP) with familial Alzheimer’s disease (FAD) causing mutations, resulting in increased lysosomal burden and a severely reduced lifespan (Centa et al., 2020). Likewise, testing in the recently developed porcine *Cln3^Δex7/8^* model (Johnson et al., 2019c) could provide important insights into efficacy in large gyrencephalic mammals.

Even with the success of the treatment in our animal models, several questions and opportunities remain. The rescues in lysosomal storage burden and neuroinflammation, while robust, are incomplete. This could be due to vector tropism patterns, timing of administration, dose, or other factors. Given the involvement of non-neuronal cell types in CLN3 disease (Xiong and Kielian, 2013; Parviainen et al., 2017), it is possible that broader and more efficient transduction, or even development of a much needed cross-correction strategy for transmembrane transgene products, could further enhance efficacy. Additionally, while we administered virus at the neonatal time point of P1, cellular defects have been observed in *Cln3^Δex7/8^* mice even before birth (Cotman et al., 2002), and such early initiation of disease cascades may be difficult to completely rescue even with optimal transduction. It is unknown whether similar early presymptomatic pathology exists in humans, but it is likely that the ideal gene therapy strategy will correct CLN3 deficiency at the earliest time point possible. If prenatal testing for CLN3 were to be widely adopted, human patients could potentially be treated before the onset of symptoms or even in utero.

In any case, the benefits provided by neonatal CSF-delivered scAAV9.Mecp2.CLN3 far surpass those offered by other treatments to date. Our results demonstrate that scAAV9.Mecp2.CLN3 provides substantial benefits to histopathological and behavioral parameters in a mouse model of CLN3 disease that recapitulates the most common mutation observed in human patients. Together with safety studies, our data contributed to the approval of a new investigational drug (IND) application and the initiation of the corresponding ongoing clinical trial Phase I/II clinical trial (NCT03770572).

## Materials and methods

Ethics statement/Animals. Wild type and homozygous *Cln3^Δ7/8^* mice developed on C57BL/6 backgrounds were used for all studies and were housed under identical conditions in an AAALAC accredited facility in accordance with IACUC approval (Sanford Research, Sioux Falls, SD).

Virus Production. A human *CLN3* cDNA clone was obtained from Origene (Rockville, MD, USA) and subcloned into an AAV production vector under the control of a truncated Mecp2 promoter. Self-complementary AAV9. Mecp2. CLN3 was produced by SAB Tech (Philadelphia, PA, USA) according to their established protocols. Purity and titer of the virus was assessed by silver staining and qPCR analysis.

scAAV9.Mecp2.CLN3 delivery. At P0 or P1, *Cln3^Δ7/8^* pups were sexed then randomly assigned to treatment groups of either PBS-treated or scAAV9.Mecp2.CLN3-treated until both treatment groups were filled. The *Cln3^Δ7/8^* mice received single P1 4 µl intracerebroventricular (ICV) injections of either PBS or scAAV9.Mecp2.CLN3 at a dose of 2.2 × 10^10^ vg/animal, following hypothermia sedation. All mice were monitored until fully recovered. Genotyping was performed using previously described techniques (Morgan et al., 2013), and all animals were monitored daily by trained animal technicians.

hCLN3 Detection. For RT-qPCR analysis, mice were CO_2_ euthanized and a 3 mm parasagittal section of the outer right hemisphere was frozen for RNA isolation. Total RNA was extracted with the Maxwell 16 LEV simplyRNA Tissue Kit (AS1280), according to the manufacturer’s instructions, using the Maxwell 16 MDx machine (Promega; Madison, WI, USA). RNA quality and concentration was assayed using a BioTek Epoch Microplate Spectrophotometer (Winooski, VT, USA), and only samples with concentrations between 200–1,000 ng/μl and an A260/A280 > 2 were used. cDNA synthesis was performed on 1 μg of total RNA using the Promega GoScript Reverse Transcription System (A5001) according to the manufacturer’s protocol. The following primers were used: hCLN3 Forward: CGC​TAG​CAT​CTC​ATC​AGG​CCT​TG; Reverse: AGC​ATG​GAC​AGC​AGG​GTC​TG. Gapdh Forward: ACC​ACA​GTC​CAT​GCC​ATC​AC; Reverse: ACC​ACA​GTC​CAT​GCC​ATC​AC. Reactions were run on an Applied Biosystems ABI 7500 Fast Real-time PCR System (Foster City, CA, USA) utilizing a touchdown-PCR protocol optimized for amplification of human *CLN3* cDNA. PCR products were run on 3% Tris-borate-EDTA gels at 125 V for 90 min. Relative levels of *hCLN3* transcript were normalized against *Gapdh* by 2^−ΔΔCt^ calculation from means of triplicate Ct values for both transcripts.

For RNAScope analysis, brains were collected from CO_2_ euthanized mice and placed on a 1 mm sagittal brain block. Tissue blocks from 0 to 3 mm right of the midline were flash frozen and sliced on a cryostat at 16 μm, series dehydrated, placed on slides, and processed for RNAScope according to the manufacturer’s protocols (Advanced Cell Diagnostics; Newark, CA, USA). Sections were labeled with a human CLN3 probe (ACDBio #497591), counterstained with DAPI, and mounted on slides using antifade mounting media (Dako faramount, Agilent).

Immunohistochemistry. Following collection of brains for RNAScope, the remaining left hemisphere of the brain was fixed in 4% paraformaldehyde and sectioned into 50 μm slices with selected slices placed into a 24 well plate. Immunohistochemistry was performed on free-floating sections as previously described using anti-hCLN3 (produced by ProSci, Poway, CA, USA; validated by Amicus Therapeutics, Philadelphia, PA, USA), anti-ATP synthase subunit C (Abcam, ab181243), anti-GFAP (Dako, Z0334), and anti-CD68 (AbD Serotec, MCA 1957) antibodies (Weimer et al., 2007; Weimer et al., 2009). Secondary antibodies included anti-rat and anti-rabbit biotinylated (Vector Labs, BA-9400). Sections were placed on slides and mounted using xylene and xylene-based mounting media (DPX, VWR International). Sections were imaged and analyzed using an Aperio Digital Pathology Slide Scanner (VERSA; Leica Biosystems; Wetzlar, Germany) and associated software. Multiple images of each animal were taken in each brain region examined and labeling intensities were quantified using ImageJ.

### Behavioral testing

Rotarod. Animals participated in an accelerated rotarod protocol as previously described to assess motor coordination from 2 to 24 months of age (Columbus Instruments; Columbus, OH, USA) (Cain et al., 2019). The machine was set to accelerate 0.3 rpm every 2 seconds, with a starting speed of 0.3 rpm and a maximum speed of 36 rpm. Mice were trained for three consecutive trials, given a 30 min rest period, trained for three consecutive trials, given a second 30 min rest period, and trained for three final consecutive trials. After a 4-h rest period, mice were tested using the same paradigm as the training session. The latency time to fall from the rod was averaged from each of the nine afternoon testing sessions to produce one value per mouse.

Pole Climb. The pole climb descent test was performed as previously described from 2 to 24 months of age (Kovacs and Pearce, 2015). Briefly, mice were placed downward on a metal pole and given 60 s to descend the pole. Mice were then placed upward on the metal pole and given 60 s to turn downward on the pole. In addition to recording latency to climb down and turn down, the number of falls made by each mouse during the two tests was recorded.

Water Maze. Mice were trialed in a four-foot diameter Morris water maze apparatus to assess memory and learning deficiencies as previously described from 2 to 24 months of age (Cain et al., 2019). Water filled the apparatus to approximately 26 inches and the goal platform was submerged by 0.5 cm at a location approximately 315° from the starting location. Four distinct visual cues were placed around the tub at 0, 90, 180, and 270° to aid in spatial memory. Mice were first trained in a clear pool with a flagged platform to rule out confounding factors such as visual and motor deficits. Mice were given 60 s to find the platform each trial, with four trials in the morning, followed by a 3-h rest period, and four additional trials in the afternoon. Mice that could not locate the platform with 50% accuracy in the time allotted were eliminated from further testing. Mice were then tested in water colored with white, non-toxic tempera paint with and an un-flagged platform. Mice were given 60 s to complete each trial, with four trials in the morning, followed by a 3-h rest period, followed by four additional trials in the afternoon. Mice were tested for four consecutive days, each day starting at a different visual cue. Mice were recorded using Any-maze video tracking software (Stoelting Co.; Wood Dale, IL, USA). Results were compiled as the average from sixteen afternoon trials per mouse.

Three Component Test. Tests were performed as previously described in 12, 14, 16, 18, and 24 month-old animals (Guyenet et al., 2010). Briefly, for hind limb clasping measurements, animals were scored based on the extent to which their limbs clasped into their abdomen when held by the base of their tail (score 0–3). For ledge lowering measurements, animals were scored based on their ability to easily lower themselves from the edge of their home cage (score 0–3). For gait measurements, animals were scored based on their overall ease of walking, including whether their abdomen dragged on the ground and if their limbs were splayed out while walking (score 0–3). For all scores, the same blinded observer recorded all measurements.

Force Plate. A force plate actimeter was used to measure motor ability as previously described in 12, 14, 16, 18, and 24 month-old animals (Geraets et al., 2017). Animals were recorded in a sound-proof chamber for 20 min, and data was processed using FPA Analysis Software (BASi, West Lafayette, IN, USA).

Running Wheel. Circadian activity patterns were assessed in 10-month old animals using a running wheel apparatus. The first experiment was conducted on a standard light/dark cycle (14 h light, 10 h dark), while the second experiment was conducted in 24 h of darkness to assess an animal’s natural circadian rhythm without light cues. Mice were singly housed in cages that held a low-profile wireless running wheel (Cat. # MED-047, Med Associates, Inc.; Georgia, VT, USA), with food and water provided ad libitum. Mice were allowed to acclimate to the running wheel for 48 h prior to data acquisition, and for the 24 h dark cycles, accommodations were made to ensure no light entered from under the door of the room or from other electronic devices in the room. Data was then acquired for a 12 day timespan, during which experimenters limited room entry to once a day for welfare checks to minimize animal disturbance. No cage changes occurred during the 2 week period, and water bottles were changed once by lab staff. For the second experiment in complete darkness, a paper log was used to record any staff or experimenter entering and exiting the dark room, and data collection was paused during these disturbance periods. Additionally, a red light was used in the room for animal and software checks as necessary. Data from both experiments were analyzed and exported using the wheel analysis software provided by Med Associates, Inc. Activity was defined as any 15-min interval with 60 or more rotations, and an animal was defined as ‘being active’ if there were at least three active periods within a 1-h period. Rest was defined as any 15-min interval with 59 or fewer rotations, and an animal was defined as ‘being at rest’ if there were at least three rest periods within a 1-h period.

Optokinetic Tracking. At 8 months of age mice were assayed in an OptoMotry optokinetic tracking chamber (Cerebral Mechanics, Inc., Medicine Hat, Alberta, Canada) as previously described (Douglas et al., 2005). Briefly, the chamber consisted of four computer monitors facing the animal, displaying a rotating black and white contrast gradient. Blinded experimenters observed whether the animal appeared to see the gradient by intentional head tracking, and scored the animal accordingly using Cerebral Mechanics software. Visual acuity was averaged from both eyes in cycles/degree.

Neonatal Behavior. Neurodevelopment was assessed as previously described from postnatal day three to 15 (Osorio et al., 2009). Animals were assessed for negative geotaxis (ability to turn upward on a 45° plane from a downward position), postural reflex (ability to maintain position when environment is shaken), paw grasping (ability to grasp a metal bar with forepaws), and exploratory behavior (ability to leave a Petri dish).

Statistical Analysis. Statistical analyses were performed using GraphPad Prism (v6.04 or later; San Diego, CA, USA). Details of the specific tests are noted in the figure legends and detailed n are found in Supplementary Table S2. Outliers were removed with the ROUT method, Q = 1%. *^/#^p < 0.05, **^/##^p < 0.01, ***^/###^p < 0.001, ****^/####^p < 0.0001.

## Data Availability

The raw data supporting the conclusions of this article will be made available by the authors, without undue reservation.
