# Supplementary material for: Early postnatal administration of an AAV9 gene therapy is safe and efficacious in CLN3 disease
Source: Front Genet. 2023 Mar 24;14:1118649. doi: 10.3389/fgene.2023.1118649 (PMC10080320; doi:10.3389/fgene.2023.1118649)
Supplement: Supplementary file 1 [file Presentation1.pdf]

Supplementary Material

Supplemental Figure 1: *hCLN3* is stably expressed throughout the mouse brain. Neonatal intracerebroventricular administration of *scAAV9.Mecp2.CLN3* results in efficient and stable expression of *hCLN3* transcript throughout the mouse brain, from 6-24 months of age. Please note that each time point was run independently of one another and cannot be directly compared to one another.

Supplemental Figure 2: No robust differences in complete blood count parameters between wild type and *Cln3*$^{\Delta ex7/8}$ mice. Measurements include: red blood cell count (A), hemoglobin (B), hematocrit (C), mean corpuscular volume (D), mean corpuscular hemoglobin (E), mean corpuscular hemoglobin concentration (F), red blood cell distribution width (G), platelets (H), mean platelet volume (I), white blood cell count (J), lymphocytes (K), monocytes (L), and granulocytes (M). One-way ANOVA at each time point. *p<0.05. Detailed Ns in Supplemental Table 2.

Supplemental Figure 3: *scAAV9.Mecp2.CLN3* prevents weight loss and hyperactivity in *Cln3*$^{\Delta ex7/8}$ mice. *scAAV9.Mecp2.CLN3* prevented *Cln3*$^{\Delta ex7/8}$ weight loss at 24 months of age (A) and hyperactivity at 18-24 months of age as measured by distance traveled (B), area traveled (C), bouts of low mobility (D), and number of focused stereotypy events (E). Two-way ANOVA, Tukey correction. Asterisks denote comparison to wild type unless otherwise noted. Hash signs denote comparison to all other groups. *p<0.05, **p<0.01, ***p<0.001, ****p<0.0001. #p<0.05, ##p<0.01, ###p<0.001, ####p<0.0001. Detailed Ns in Supplemental Table 2.

Supplemental Figure 4: No differences in neonatal behaviors were detected between wild type and *Cln3*$^{\Delta 7/8}$ mice. When assessing neonatal behaviors including adult-like negative geotaxis (A), dish test response (B), postural reflex (C), and grasping (D), we found no consistent differences in behavior between groups. Two-way ANOVA, Tukey correction. Detailed Ns in Supplemental Table 2.

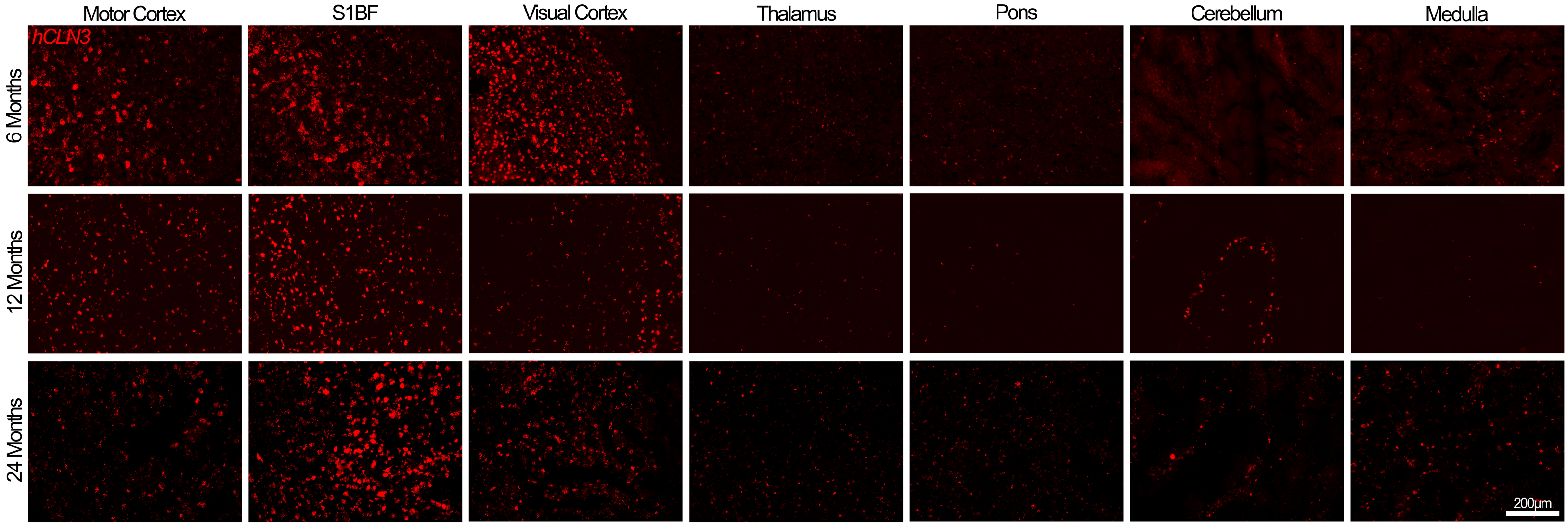
Motor Cortex
S1BF
Visual Cortex
Thalamus
Pons
Cerebellum
Medulla
hCLN3
6 Months
12 Months
24 Months
200µm

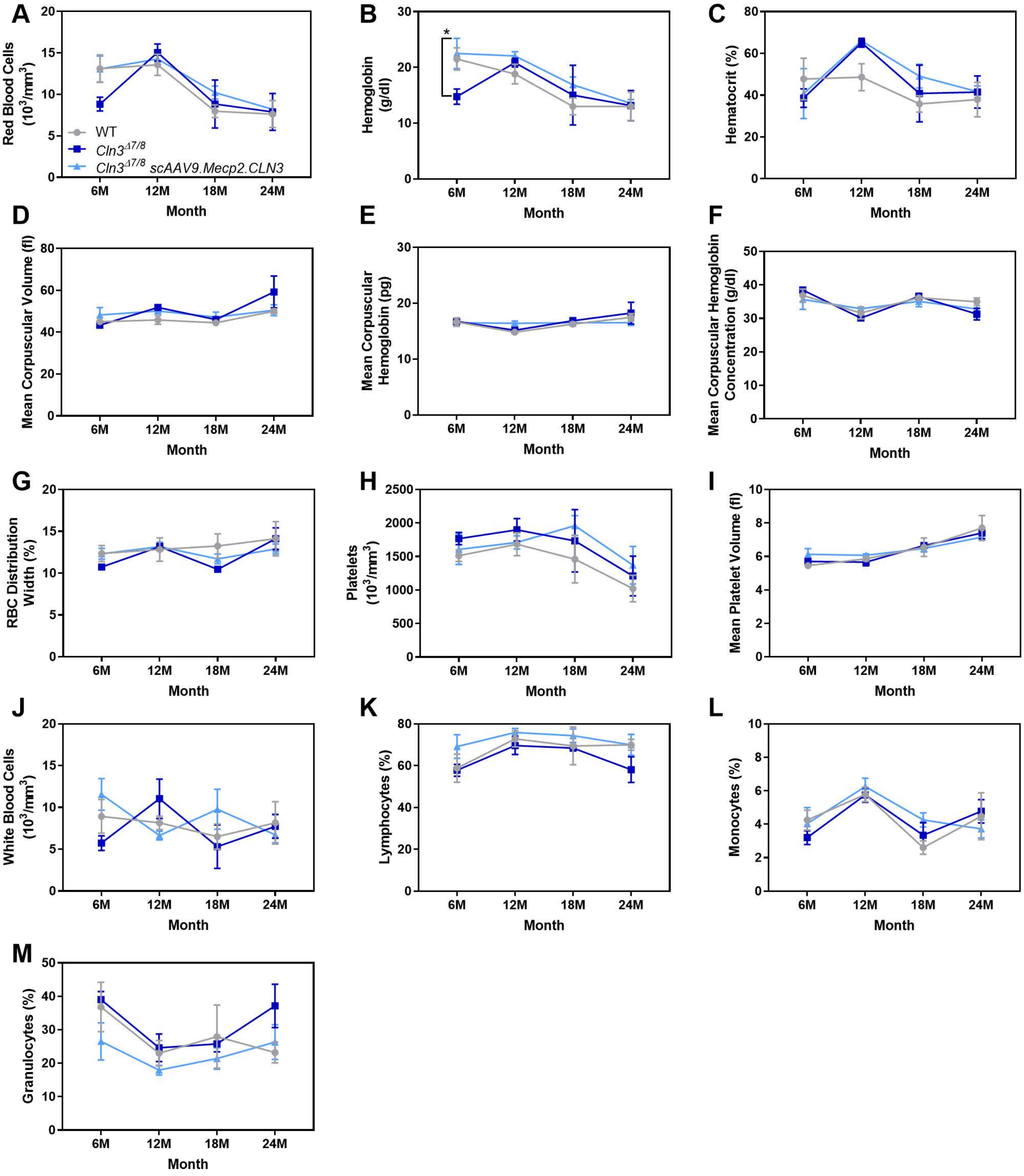
A
Red Blood Cells (10^3/mm^3)
WT
Cln3^Δ7/8
Cln3^Δ7/8 scAAV9.Mecp2.CLN3
B
Hemoglobin (g/dl)
*
C
Hematocrit (%)
D
Mean Corpuscular Volume (fl)
E
Mean Corpuscular Hemoglobin (pg)
F
Mean Corpuscular Hemoglobin Concentration (g/dl)
G
RBC Distribution Width (%)
H
Platelets (10^3/mm^3)
I
Mean Platelet Volume (fl)
J
White Blood Cells (10^3/mm^3)
K
Lymphocytes (%)
L
Monocytes (%)
M
Granulocytes (%)
6M
12M
18M
24M
Month

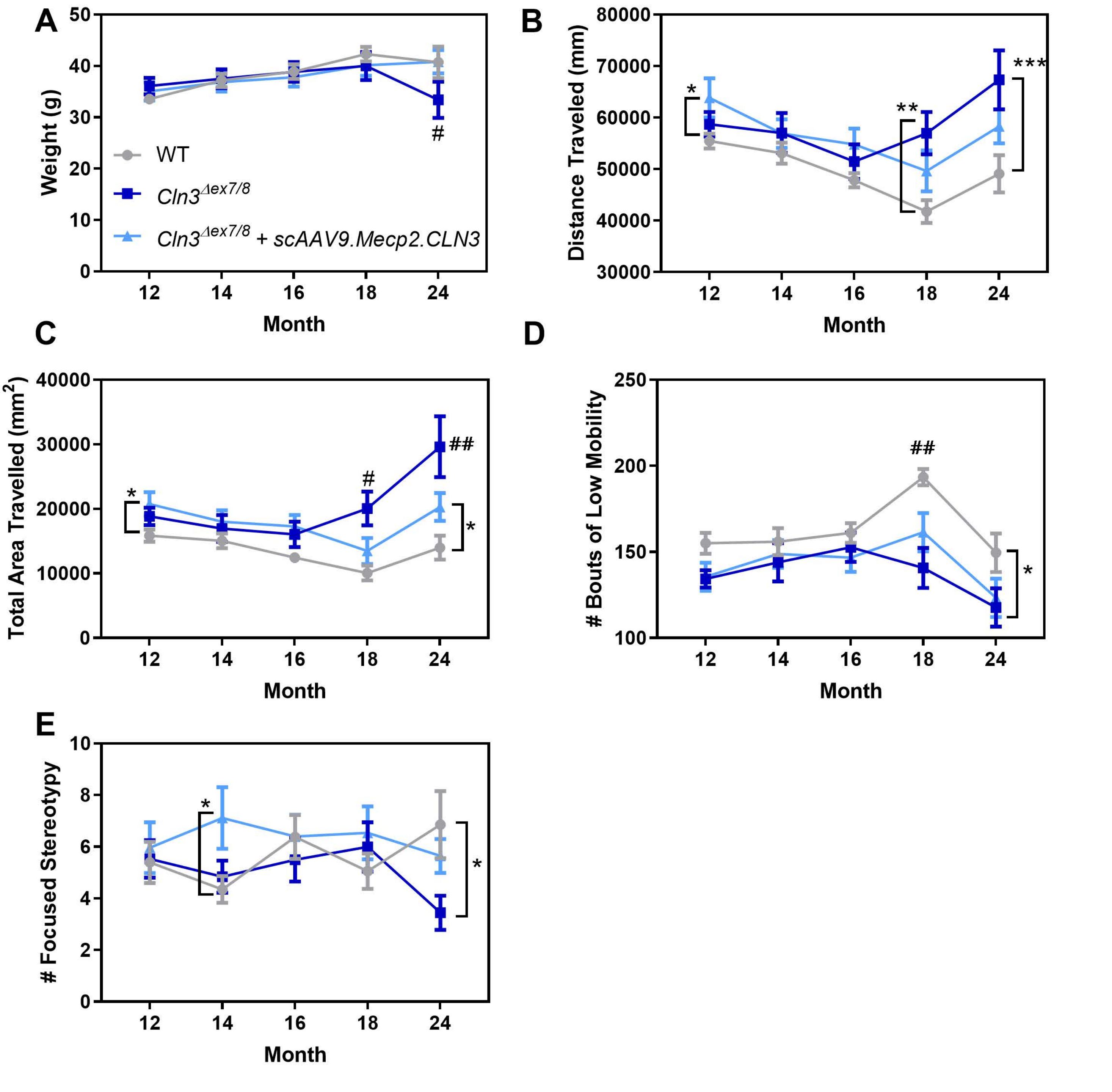

A
Weight (g)
#
WT
Cln3Δex7/8
Cln3Δex7/8 + scAAV9.Mecp2.CLN3
Month
B
Distance Traveled (mm)
*
**
***
Month
C
Total Area Travelled (mm2)
*
#
##
*
Month
D
Bouts of Low Mobility
##
*
Month
E
Focused Stereotypy
*
*
Month

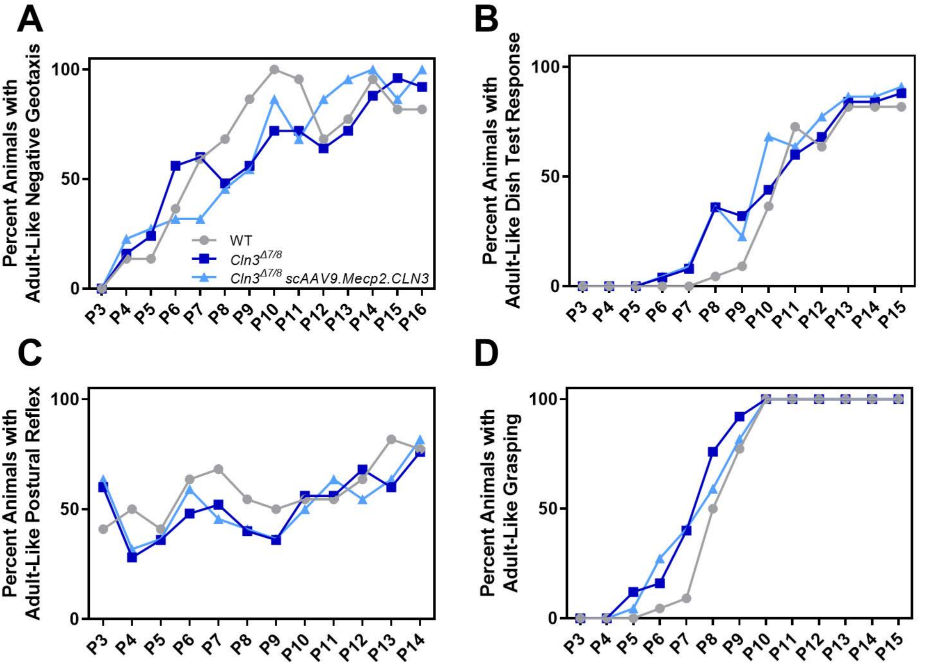
A
Percent Animals with Adult-Like Negative Geotaxis
WT
$Cln3^{\Delta 7/8}$
$Cln3^{\Delta 7/8}$ scAAV9.Mecp2.CLN3
B
Percent Animals with Adult-Like Dish Test Response
C
Percent Animals with Adult-Like Postural Reflex
D
Percent Animals with Adult-Like Grasping

Supplemental Table 1. Death and euthanasia descriptions.

Treatment Group	Sex	Age at Death	Morbidity Notes
Cln3$^{Δ7/8}$	F	14	Found dead
Cln3$^{Δ7/8}$	F	18	Euthanized - unhealing wound
Cln3$^{Δ7/8}$	F	20	Euthanized - unhealing wound and ulcerative dermatitis
Cln3$^{Δ7/8}$	F	21	Euthanized - Mass on shoulder/throat; labored breathing
Cln3$^{Δ7/8}$	F	22	Found dead
Cln3$^{Δ7/8}$	F	24	n/a - retired from study
Cln3$^{Δ7/8}$	F	24	n/a - retired from study
Cln3$^{Δ7/8}$	F	24	n/a - retired from study
Cln3$^{Δ7/8}$	F	24	n/a - retired from study
Cln3$^{Δ7/8}$	F	24	n/a - retired from study
Cln3$^{Δ7/8}$	F	24	n/a - retired from study
Cln3$^{Δ7/8}$	F	24	n/a - retired from study
Cln3$^{Δ7/8}$	F	24	n/a - retired from study
Cln3$^{Δ7/8}$	F	24	n/a - retired from study
Cln3$^{Δ7/8}$	F	24	n/a - retired from study
Cln3$^{Δ7/8}$	M	13	Found dead
Cln3$^{Δ7/8}$	M	15	Found dead
Cln3$^{Δ7/8}$	M	18	Found dead
Cln3$^{Δ7/8}$	M	24	n/a - retired from study
Cln3$^{Δ7/8}$	M	24	n/a - retired from study
Cln3$^{Δ7/8}$	M	24	n/a - retired from study
Cln3$^{Δ7/8}$	M	24	n/a - retired from study
Cln3$^{Δ7/8}$	M	24	n/a - retired from study
Cln3$^{Δ7/8}$	M	24	n/a - retired from study
Cln3$^{Δ7/8}$	M	24	n/a - retired from study
Cln3$^{Δ7/8}$	M	24	n/a - retired from study
Cln3$^{Δ7/8}$	M	24	n/a - retired from study
Cln3$^{Δ7/8}$	M	24	n/a - retired from study
Cln3$^{Δ7/8}$ + AAV9	F	5	Euthanized - Moribund; hunched
Cln3$^{Δ7/8}$ + AAV9	F	19	Found dead
Cln3$^{Δ7/8}$ + AAV9	F	21	Euthanized - Mass L side of face
Cln3$^{Δ7/8}$ + AAV9	F	22	Found dead
Cln3$^{Δ7/8}$ + AAV9	F	22	Found dead
Cln3$^{Δ7/8}$ + AAV9	F	24	Euthanized - unhealing wound
Cln3$^{Δ7/8}$ + AAV9	F	24	n/a - retired from study. Noted poor body condition, significant anal prolapse
Cln3$^{Δ7/8}$ + AAV9	F	24	n/a - retired from study; large abscess noted on L shoulder
Cln3$^{Δ7/8}$ + AAV9	F	24	n/a - retired from study
Cln3$^{Δ7/8}$ + AAV9	F	24	n/a - retired from study
Cln3$^{Δ7/8}$ + AAV9	F	24	n/a - retired from study
Cln3$^{Δ7/8}$ + AAV9	F	24	n/a - retired from study
Cln3$^{Δ7/8}$ + AAV9	F	24	n/a - retired from study
Cln3$^{Δ7/8}$ + AAV9	F	24	n/a - retired from study
Cln3$^{Δ7/8}$ + AAV9	M	4	Euthanized - Moribund; Bladder contained 15uL of urine, possible urethral obstruction
Cln3$^{Δ7/8}$ + AAV9	M	23	Found dead
Cln3$^{Δ7/8}$ + AAV9	M	24	Found dead
Cln3$^{Δ7/8}$ + AAV9	M	24	n/a - retired from study
Cln3$^{Δ7/8}$ + AAV9	M	24	n/a - retired from study
Cln3$^{Δ7/8}$ + AAV9	M	24	n/a - retired from study
Cln3$^{Δ7/8}$ + AAV9	M	24	n/a - retired from study
Cln3$^{Δ7/8}$ + AAV9	M	24	n/a - retired from study
Cln3$^{Δ7/8}$ + AAV9	M	24	n/a - retired from study; hydrocephaly noted during necropsy
Cln3$^{Δ7/8}$ + AAV9	M	24	n/a - retired from study
Cln3$^{Δ7/8}$ + AAV9	M	24	n/a - retired from study
Cln3$^{Δ7/8}$ + AAV9	M	24	n/a - retired from study

Treatment Group	Sex	Age at Death	Morbidity Notes
Cln3$^{Δ7/8}$ + AAV9	M	24	n/a - retired from study
Cln3$^{Δ7/8}$ + AAV9	M	24	n/a - retired from study
WT	F	3	Found dead
WT	F	18	Found dead
WT	F	19	Euthanized - unhealing wound around R eye
WT	F	20	Found dead
WT	F	20	Euthanized - unhealing wound
WT	F	24	n/a - retired from study
WT	F	24	n/a - retired from study
WT	F	24	n/a - retired from study
WT	F	24	n/a - retired from study
WT	F	24	n/a - retired from study
WT	F	24	n/a - retired from study
WT	F	24	n/a - retired from study
WT	F	24	n/a - retired from study
WT	F	24	n/a - retired from study
WT	F	24	n/a - retired from study
WT	M	22	Euthanized - Severe head tilt; unable to right itself
WT	M	23	Euthanized - Penile prolapse, ulceritive dermatitis
WT	M	23	Found dead
WT	M	23	Euthanized - Hunched, swollen abdomen, rectal prolapse
WT	M	23	Euthanized - Labored breathing; hunched
WT	M	23	Euthanized - Edema, hunching
WT	M	24	n/a - retired from study
WT	M	24	n/a - retired from study
WT	M	24	n/a - retired from study
WT	M	24	n/a - retired from study
WT	M	24	n/a - retired from study
WT	M	24	n/a - retired from study
WT	M	24	n/a - retired from study
WT	M	24	n/a - retired from study; some seizures noted in last month of life

Supplemental Table 2. Detailed animal n for each experiment (n=number of animals). All groups are mixed and equal sexes unless otherwise specified.

		WT										*Cln3*$^{\Delta 7/8}$										*Cln3*$^{\Delta 7/8}$+ AAV9									
		2	4	6	8	10	12	14	16	18	24	2	4	6	8	10	12	14	16	18	24	2	4	6	8	10	12	14	16	18	24
qPCR	Cerebral Cortex	6	6	12	6	6	4	-	-	5	5	6	6	10	5	5	6	-	-	6	6	6	4	8	6	7	6	-	-	6	6
	Cervical SC	6	5	12	5	6	5	-	-	5	6	6	5	12	5	4	6	-	-	6	5	6	4	8	6	7	6	-	-	6	8
	Thoracic SC	6	6	6	5	6	5	-	-	4	5	6	6	6	6	4	6	-	-	6	4	6	4	4	5	6	6	-	-	6	7
	Lumbar SC	6	6	6	5	6	4	-	-	4	5	6	6	6	5	5	6	-	-	5	4	6	4	4	6	7	6	-	-	5	7
	Kidney	6	6	5	5	6	5	-	-	5	5	6	6	5	6	5	6	-	-	6	6	6	4	4	6	7	5	-	-	4	7
hCLN3	S1BF	-	-	5	-	-	-	-	-	5	-	-	-	6	-	-	-	-	-	6	-	-	-	5	-	-	-	-	-	7	-
	VPM/VPL	-	-	2	-	-	-	-	-	5	-	-	-	2	-	-	-	-	-	6	-	-	-	3	-	-	-	-	-	7	-
	CA3	-	-	2	-	-	-	-	-	5	-	-	-	2	-	-	-	-	-	6	-	-	-	3	-	-	-	-	-	7	-
ASM	S1BF	6	6	5	5	5	6	-	-	5	4	6	6	4	6	6	5	-	-	6	8	6	6	6	6	7	6	-	-	6	8
	VPM/VPL	6	6	5	6	5	6	-	-	5	4	6	6	4	6	6	5	-	-	6	8	6	6	6	5	7	6	-	-	6	8
SubC	S1BF	5	5	4	5	6	6	-	-	6	6	5	6	5	5	6	6	-	-	6	6	5	6	6	7	6	6	-	-	6	7
	VPM/VPL	5	6	5	5	5	6	-	-	6	6	5	6	5	6	6	6	-	-	6	6	5	6	6	7	6	4	-	-	6	7
	BL-Amygdala, Male	-	-	-	-	-	3	-	-	3	-	-	-	-	-	-	3	-	-	3	-	-	-	-	-	-	3	-	-	3	-
	BL-Amygdala, Female	-	-	-	-	-	3	-	-	2	-	-	-	-	-	-	3	-	-	3	-	-	-	-	-	-	3	-	-	3	-
	CA3, Male	-	-	-	-	-	3	-	-	3	-	-	-	-	-	-	3	-	-	3	-	-	-	-	-	-	3	-	-	3	-
	CA3, Female	-	-	-	-	-	3	-	-	2	-	-	-	-	-	-	3	-	-	3	-	-	-	-	-	-	3	-	-	3	-
	DG, Male	-	-	-	-	-	3	-	-	3	-	-	-	-	-	-	3	-	-	3	-	-	-	-	-	-	2	-	-	3	-
	DG, Female	-	-	-	-	-	2	-	-	2	-	-	-	-	-	-	2	-	-	2	-	-	-	-	-	-	3	-	-	3	-
	MD-Thal, Male	-	-	-	-	-	3	-	-	3	-	-	-	-	-	-	3	-	-	3	-	-	-	-	-	-	2	-	-	3	-
	MD-Thal, Female	-	-	-	-	-	3	-	-	2	-	-	-	-	-	-	3	-	-	3	-	-	-	-	-	-	3	-	-	3	-
	Piriform, Male	-	-	-	-	-	3	-	-	3	-	-	-	-	-	-	3	-	-	3	-	-	-	-	-	-	3	-	-	3	-
	Piriform, Female	-	-	-	-	-	3	-	-	2	-	-	-	-	-	-	3	-	-	3	-	-	-	-	-	-	3	-	-	3	-
	S1BF, Male	-	-	-	-	-	3	-	-	3	-	-	-	-	-	-	3	-	-	3	-	-	-	-	-	-	3	-	-	3	-
	S1BF, Female	-	-	-	-	-	3	-	-	2	-	-	-	-	-	-	3	-	-	3	-	-	-	-	-	-	3	-	-	3	-
	VPM/VPL, Male	-	-	-	-	-	3	-	-	3	-	-	-	-	-	-	3	-	-	3	-	-	-	-	-	-	2	-	-	3	-
	VPM/VPL, Female	-	-	-	-	-	3	-	-	2	-	-	-	-	-	-	3	-	-	3	-	-	-	-	-	-	3	-	-	3	-
	Ret Thal, Male	-	-	-	-	-	3	-	-	3	-	-	-	-	-	-	3	-	-	3	-	-	-	-	-	-	3	-	-	3	-
	Ret Thal, Female	-	-	-	-	-	3	-	-	2	-	-	-	-	-	-	3	-	-	3	-	-	-	-	-	-	3	-	-	3	-
GFAP	S1BF	6	6	3	6	5	5	-	-	5	6	6	6	6	6	6	6	-	-	6	6	6	6	6	6	6	6	-	-	6	6
	VPM/VPL	6	6	5	6	5	5	-	-	6	5	6	6	6	6	6	6	-	-	6	6	6	6	6	6	6	4	-	-	5	6
CD68	S1BF	6	5	4	6	6	5	-	-	5	4	6	6	6	6	6	6	-	-	6	6	6	6	6	6	6	6	-	-	6	4
	VPM/VPL	6	6	6	6	5	5	-	-	6	3	6	6	8	6	6	6	-	-	6	6	6	6	6	6	6	6	-	-	6	4
	MD-Thal, Male	-	-	-	-	-	2	-	-	3	-	-	-	-	-	-	3	-	-	3	-	-	-	-	-	-	3	-	-	3	-
	MD-Thal, Female	-	-	-	-	-	3	-	-	2	-	-	-	-	-	-	3	-	-	3	-	-	-	-	-	-	3	-	-	3	-
	SM-Thal, Male	-	-	-	-	-	2	-	-	2	-	-	-	-	-	-	3	-	-	3	-	-	-	-	-	-	3	-	-	3	-
	SM-Thal, Female	-	-	-	-	-	3	-	-	2	-	-	-	-	-	-	3	-	-	2	-	-	-	-	-	-	3	-	-	3	-
	VPM/VPL, Male	-	-	-	-	-	2	-	-	3	-	-	-	-	-	-	3	-	-	3	-	-	-	-	-	-	3	-	-	3	-
	VPM/VPL, Female	-	-	-	-	-	3	-	-	2	-	-	-	-	-	-	3	-	-	3	-	-	-	-	-	-	3	-	-	3	-
	S1BF, Male	-	-	-	-	-	2	-	-	3	-	-	-	-	-	-	3	-	-	3	-	-	-	-	-	-	3	-	-	3	-
	S1BF, Female	-	-	-	-	-	3	-	-	2	-	-	-	-	-	-	3	-	-	3	-	-	-	-	-	-	3	-	-	3	-
Pole	Climb Down	22	20	20	20	20	18	20	20	18	11	20	20	19	19	19	19	18	18	18	10	22	19	19	19	18	19	19	19	19	13
	Turn	22	20	20	20	20	20	20	20	18	11	20	20	19	19	19	19	18	18	18	11	22	19	19	19	19	19	19	19	19	12

		WT										$Cln3^{\Delta 7/8}$										$Cln3^{\Delta 7/8}$+ AAV9									
		2	4	6	8	10	12	14	16	18	24	2	4	6	8	10	12	14	16	18	24	2	4	6	8	10	12	14	16	18	24
	# Falls	22	20	20	20	20	20	20	20	18	11	20	20	19	19	18	19	18	19	18	11	22	18	19	19	19	19	19	19	19	13
Maze	Time	19	16	18	18	18	18	18	18	14	9	18	20	19	17	18	17	17	17	17	7	18	16	15	15	15	14	16	15	15	8
	Speed	20	18	18	18	18	18	18	17	16	9	18	20	19	18	18	17	17	17	17	7	18	18	17	17	15	14	17	17	16	8
Force Plate	Weight	-	-	-	-	-	21	21	20	20	11	-	-	-	-	-	18	17	18	13	11	-	-	-	-	-	19	19	18	19	14
	Total Distance	-	-	-	-	-	21	21	20	18	11	-	-	-	-	-	18	17	18	13	12	-	-	-	-	-	19	19	18	19	14
	Total Area	-	-	-	-	-	21	12	20	18	11	-	-	-	-	-	18	17	18	13	12	-	-	-	-	-	19	19	18	19	14
	Bouts Low Mobility	-	-	-	-	-	21	21	20	17	11	-	-	-	-	-	18	17	17	13	12	-	-	-	-	-	19	19	18	19	14
	Stereotypy	-	-	-	-	-	20	20	20	18	11	-	-	-	-	-	18	17	17	13	12	-	-	-	-	-	19	19	18	18	14
Other Behavior	Rotarod	22	20	20	20	20	20	20	20	18	11	20	20	19	19	19	19	18	18	18	10	21	20	19	19	19	19	19	19	19	10
	Coordination Score	-	-	-	-	-	20	20	20	18	11	-	-	-	-	-	20	19	19	18	12	-	-	-	-	-	18	18	19	19	13
	Optokinetic Track	-	-	-	13	-	-	-	-	-	-	-	-	-	15	-	-	-	-	-	-	-	-	-	18	-	-	-	-	-	-
	Running Wheel	-	-	-	-	20	-	-	-	-	-	-	-	-	-	20	-	-	-	-	-	-	-	-	-	20	-	-	-	-	-
	Neonatal Develop	22 (Neonatal)										25 (Neonatal)										22 (Neonatal)									
	Survival	-	-	-	-	-	-	-	-	-	29	-	-	-	-	-	-	-	-	-	28	-	-	-	-	-	-	-	-	-	28
CBCs	RBC	-	-	4	-	-	6	2	5	-	-	3	-	-	6	2	4	-	-	5	-	-	6	5	4		6	-	-	4	-
	HGB	-	-	4	-	-	5	-	-	2	5	-	-	3	-	-	5	-	-	2	4	-	-	5	-	-	4	-	-	5	4
	HCT	-	-	2	-	-	4	-	-	2	5	-	-	3	-	-	3	-	-	2	4	-	-	2	-	-	3	-	-	5	4
	MCV	-	-	4	-	-	6	-	-	2	5	-	-	3	-	-	6	-	-	2	4	-	-	5	-	-	6	-	-	5	4
	MCH	-	-	4	-	-	6	-	-	2	5	-	-	3	-	-	6	-	-	2	4	-	-	5	-	-	6	-	-	5	4
	MCHC	-	-	4	-	-	6	-	-	2	5	-	-	3	-	-	6	-	-	2	4	-	-	5	-	-	6	-	-	5	4
	RDW	-	-	4	-	-	6	-	-	2	5	-	-	3	-	-	6	-	-	2	4	-	-	5	-	-	6	-	-	5	4
	PLT	-	-	4	-	-	5	-	-	2	5	-	-	3	-	-	5	-	-	2	4	-	-	4	-	-	6	-	-	5	4
	MPV	-	-	4	-	-	5	-	-	2	5	-	-	3	-	-	5	-	-	2	4	-	-	4	-	-	6	-	-	5	4
	WBC	-	-	4	-	-	6	-	-	2	5	-	-	3	-	-	6	-	-	2	4	-	-	5	-	-	6	-	-	5	4
	LYM	-	-	4	-	-	5	-	-	2	4	-	-	3	-	-	6	-	-	2	4	-	-	5	-	-	6	-	-	5	4
	MON	-	-	4	-	-	5	-	-	2	5	-	-	3	-	-	6	-	-	2	4	-	-	5	-	-	6	-	-	5	4
	GRA	-	-	4	-	-	6	-	-	2	5	-	-	3	-	-	6	-	-	2	4	-	-	5	-	-	6	-	-	5	4
